# *miR-210* controls the evening phase of circadian locomotor rhythms through repression of *Fasciclin 2*

**DOI:** 10.1371/journal.pgen.1007655

**Published:** 2019-07-29

**Authors:** Ye Niu, Zhenxing Liu, Xiaoge Nian, Xuehan Xu, Yong Zhang

**Affiliations:** 1 Department of Biology, University of Nevada Reno, Reno, NV, United States of America; 2 Department of Entomology and MOA Key Lab of Pest Monitoring and Green Management, College of Plant Protection, China Agricultural University, Beijing, China; Washington University in Saint Louis School of Medicine, UNITED STATES

## Abstract

Circadian clocks control the timing of animal behavioral and physiological rhythms. Fruit flies anticipate daily environmental changes and exhibit two peaks of locomotor activity around dawn and dusk. microRNAs are small non-coding RNAs that play important roles in post-transcriptional regulation. Here we identify *Drosophila miR-210* as a critical regulator of circadian rhythms. Under light-dark conditions, flies lacking *miR-210* (*miR-210*^*KO*^) exhibit a dramatic 2 hrs phase advance of evening anticipatory behavior. However, circadian rhythms and molecular pacemaker function are intact in *miR-210*^*KO*^ flies under constant darkness. Furthermore, we identify that *miR-210* determines the evening phase of activity through repression of the cell adhesion molecule *Fasciclin 2 (Fas2)*. Ablation of the *miR-210* binding site within the 3’ UTR of *Fas2* (*Fas2*^*ΔmiR-210*^) by CRISPR-Cas9 advances the evening phase as in *miR-210*^*KO*^. Indeed, *miR-210* genetically interacts with *Fas2*. Moreover, Fas2 abundance is significantly increased in the optic lobe of *miR-210*^*KO*^. In addition, overexpression of *Fas2* in the *miR-210* expressing cells recapitulates the phase advance behavior phenotype of *miR-210*^*KO*^. Together, these results reveal a novel mechanism by which *miR-210* regulates circadian locomotor behavior.

## Author summary

Circadian clocks control the timing of animal physiology. *Drosophila* has been a powerful model in understanding the mechanisms of circadian regulation. Fruit flies anticipate daily environmental changes and exhibit two peaks of locomotor activity around dawn and dusk. Here we identify *miR-210* as a critical regulator of evening anticipatory behavior. Depletion of *miR-210* in flies advances evening anticipation. Furthermore, we identify the cell adhesion molecule *Fas2* as *miR-210’s* target in circadian regulation. Fas2 abundance is increased in fly brain lacking of *miR-210*. Using CRISPR-Cas9 genome editing method, we deleted the *miR-210* binding site on the 3’ untranslated region of *Fas2* and observed similar phenotype as *miR-210* mutants. Altogether, our results indicate a novel mechanism of *miR-210* in regulation of circadian anticipatory behavior through inhibition of *Fas2*.

## Introduction

The circadian locomotor rhythms of flies are generated by a neuronal network, which consists of ~150 brain circadian neurons expressing core pacemaker genes [1–4]. These neurons can be further divided into 7 clusters based on their cell localization and neurotransmitters they express [4]. There are 3 groups of dorsal neurons (DN1, DN2, and DN3), 2 groups of ventral-lateral neurons (large and small LNv), dorsal lateral neurons (LNd), and lateral posterior neurons (LPN) [4]. The large LNvs (lLNvs) and 4 small LNvs (sLNvs) express the neuropeptide Pigment Dispersing Factor (PDF), while the 5^th^ sLNv is PDF negative. Under regular light-dark (LD) conditions, flies exhibit a bimodal pattern of locomotor rhythms, peaking around dawn and dusk, which are termed morning peak and evening peak, respectively. According to the dual-oscillator model, separate morning and evening oscillators track these behavior peaks separately [5]. Indeed, studies have shown that the PDF-positive sLNvs are responsible for promoting the morning peak, while the LNds, as well as the fifth sLNv, are mainly responsible for generating the evening activity [6, 7]. In addition, the DN1s are important to integrate environmental inputs such as light and temperature, and regulate circadian rhythms and sleep behavior [8, 9]. A recent study indicated that DN1s sense acute temperature fluctuations to modulate fly sleep [10].

The PDF positive sLNvs are the master pacemaker neurons in fly brain: they synchronize other circadian neurons to maintain robust circadian rhythms under constant darkness [11]. These sLNvs send axonal projections toward the dorsal brain region, where the DN1s and DN2s are located [12, 13]. The dorsal projections of sLNvs exhibit circadian arborization rhythms with higher complexity of axon terminals found in the early day and lower complexity at the night [14]. The physiological relevance and molecular mechanisms underlying this structural plasticity of sLNvs remain unclear. The Rho1 GTPase was identified to modulate the sLNv plasticity and affect seasonal adaptation by phosphorylating Myosion [15]. The cell adhesion molecule Fasciclin 2 (Fas2) has been found to regulate the arborization rhythms of sLNvs [16]. In addition, two matrix metalloproteinases, MMP1 and MMP2 as well as the pacemaker protein VRILLE were shown to be required for the structural remodeling control of sLNv projections [17, 18].

Circadian rhythms are generated by an intracellular molecular clock, which is conserved across the animal kingdom [19]. The core of this molecular pacemaker is a negative transcriptional-translational feedback loop [20–22]. In *Drosophila*, CLOCK (CLK) dimerizes with CYCLE (CYC) to activate rhythmic transcription of hundreds of genes through the E-box region [23, 24]. Among these clock-controlled genes, PERIOD (PER) and TIMELESS (TIM) are the key transcription repressors: they form heterodimers in the cytoplasm, and then enter into the nucleus to block their own transcription. The abundance of PER and TIM is tightly regulated by a series of post-translational modifications such as phosphorylation, glycosylation and ubiquitination [25–28]. Recent research has revealed an abundance of post-transcriptional regulation of circadian rhythms by both RNA binding proteins and miRNAs [29–31].

miRNAs are small non-coding RNAs, which repress target gene expression through mRNA degradation and/or translation inhibition, thus playing crucial roles in post-transcriptional regulation [32]. Recent studies have uncovered functions of the miRNA biogenesis pathway and specific miRNAs in regulation of different aspects of animal circadian rhythms [31, 33–35]. For instance, mouse *miR-132/212* modulates the seasonal adaptation and circadian entrainment to day length [36]. miRNAs also target the molecular clock to control period length and rhythmicity of circadian locomotor activity [37]. In *Drosophila*, the miRNA *bantam* regulates circadian locomotor period by repressing *Clk*, while *miR-276a* and *let-7* inhibit the clock genes *timeless* and *clockwork orange* to modulate circadian rhythms respectively [33, 38, 39]. Moreover, various circadian output pathways are controlled by miRNAs. The *miR959-964* cluster miRNAs regulate the circadian timing of feeding and immune response, while *miR-279* modulates circadian locomotor behavior output [40, 41]. Recently, studies have demonstrated that *miR-124* specifically controls the phase of circadian locomotor rhythms under constant darkness [42, 43].

Previously we identified that depletion of GW182, the key protein for miRNA function, affects PDF-receptor signaling and disrupts circadian rhythms [35]. To further understand the roles of miRNA, we performed a genome-wide screen for miRNAs controlling circadian rhythm phenotypes. Here we show that a conserved miRNA in animals, *miR-210*, regulates circadian rhythms in *Drosophila*. *miR-210* determines the phase of evening anticipatory behavior through inhibition of *Fas2*.

## Results

### A genetic screen of miRNA mutants identifies *miR-210* as a regulator of circadian behavior

**Fig 1:**
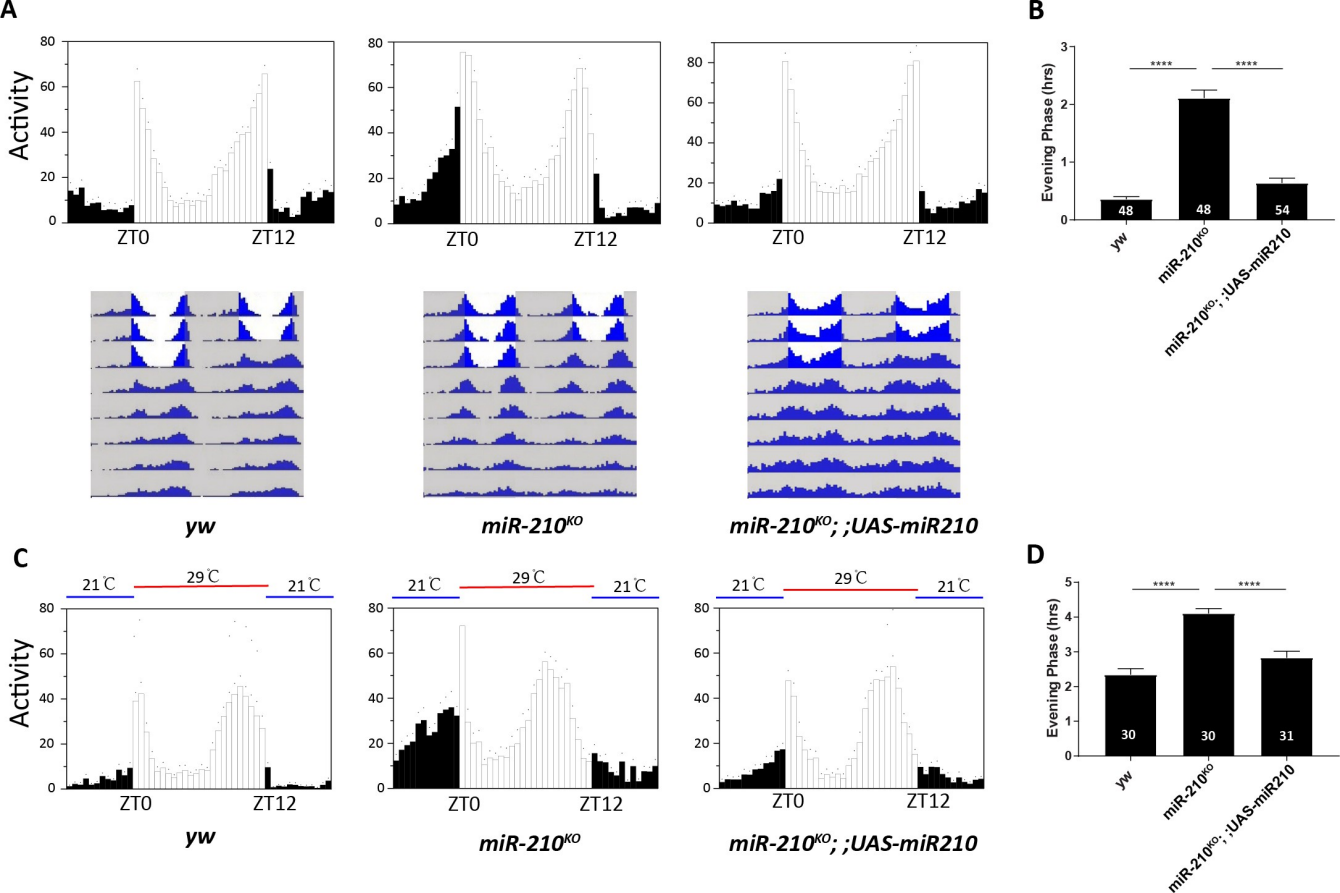
Loss of *miR-210* advances evening peak activity under LD and temperature cycles (TC). (A) Upper panel: representative eduction profiles of fly locomotor activity under 12:12 LD cycle. Black represents the dark phase, while white represents the light phase. Eduction is analyzed based on average of 3 days LD. Lower panel: representative double plotted actograms of flies under LD and DD conditions. Grey shadow represents the dark phase, while white represents the light phase. Here the knocking-in *miR-210* GAL4 (*miR-210*^*KO*^) was used to express UAS-*miR210* for rescue. (B) Quantification of evening phase of flies under LD. Phase point is determined by the time differences between the peak of evening activity and light off time. Number of flies tested is listed in each bar. Error bars indicate Standard Error. **** = p<0.0001, determined by Student’s t test. (C) Representative eduction profiles of flies under 29°C: 21°C temperature cycles (TC) in DD. Blue represents 21°C for 12 hours, while red represents 29°C. (D) Quantification of evening phase of flies under TC. Error bars represent Standard Error. **** = p<0.0001 was determined by Student’s t test.

To identify miRNAs that regulate circadian rhythms, we screened the circadian locomotor rhythms of available miRNA mutants from the Bloomington stock center. Circadian behavior of 46 lines in total was observed both under constant darkness (DD) and light dark (LD) cycles. Most of the miRNA mutants we tested exhibited normal circadian rhythms under DD; though, a few miRNAs had a slightly altered period or rhythmicity (S1 Table). Interestingly, we found that the *miR-210* mutant (*miR-210*^*KO*^) exhibits a ~1.5 hour advance of the subjective evening peak in DD (S1 Fig). We then closely examined for the locomotor behavior under LD. Indeed, we found that the *miR-210* mutant (*miR-210*^*KO*^) clearly advances the phase of the evening anticipatory peak about 2 hours (Fig 1A and 1B). The *miR-210*^*KO*^ mutant was generated by knocking-in a GAL4 cDNA in replacement of the endogenous *miR-210* sequence [44]. To rule out the possibilities of off-target or genetic background effects, we utilized the *miR-210* knock-in mutant to perform a rescue. By restoring the expression of *miR-210*, we successfully corrected the evening anticipation in the *miR-210*^*KO*^ (Fig 1A and 1B) under LD, which confirms that the phase advance is due to lack of *miR-210*. We also observed a partial but significant rescue of the phase in DD (S1 Fig).

To examine whether *miR-210* plays a role on evening anticipatory behavior under other entrainment conditions, we entrained the flies under 29°C: 21°C cycles (TC) in constant darkness. Under TC, the evening anticipation of wild-type flies exhibited an advance about 2.5 hours (Fig 1C and 1D) [45]. In *miR-210*^*KO*^, we still observed an additional 2 hrs phase advance compared to the controls (Fig 1C and 1D). Furthermore, we were able to rescue this behavior defect. After released into constant 21°C or 29°C in DD, the *miR-210*^*KO*^ flies exhibited normal period and rhythmicity as wild-type controls (S2 Table). These data confirmed that *miR-210* is a clear regulator of evening phase during different entrainment conditions.

The phase advance of evening anticipation in *miR-210*^*KO*^ is reminiscent of the *pdf* mutant phenotype [11]. To test the genetic interaction of *miR-210* and the PDF pathway, we generated *miR-210*^*KO*^; *pdf*^*01*^ double mutants. Under 12:12 LD conditions, the phase advance of the evening peak in *miR-210*^*KO*^ was indistinguishable from the *pdf*^*01*^ mutants (S2 Fig). No additive effect on the phase advance was observed in the *miR-210*^*KO*^; *pdf*^*01*^ double mutants comparing with *miR-210*^*KO*^ or *pdf*^*01*^ (S2A and S2B Fig). To observe the phenotype even more clearly, we tested these flies under long photoperiods with 16:8 LD cycle so that we avoid acute effects of the light-off transition on the shape of the evening peak. As observed in *pdf*^*01*^ mutant, *miR-210*^*KO*^ advanced the phase of evening anticipation by about 2 hrs (S2C Fig). Again, no additive phase advance was observed in the *miR-210*^*KO*^; *pdf*^*01*^ double mutants (S2D Fig). These data suggest that *miR-210* functions in the same pathway as PDF in regulation of evening phase under entrainment.

To further test the role of *miR-210*, we first overexpressed it in all circadian tissues using *tim-GAL4* [46]. However, most of the flies with overexpression died rapidly after the LD cycles, which indicates that overexpression leads to severe health issues (Table 1). Thus, we then used the *pdf-GAL4* to specifically drive *miR-210* expression in PDF positive pacemaker neurons. The majority of flies (~63%) with *miR-210* overexpression in PDF neurons became arrhythmic under DD. However, for the 37% rhythmic flies, the circadian period was lengthened about 2 hours. Thus, while loss of *miR-210* has no effect on the period and amplitude of circadian rhythms in DD, overexpression is detrimental to the proper function of the circadian oscillator in PDF neurons.

**Table 1:** Locomotor activity of flies with altered *miR-210* and *Fas2* in DD.

Genotype	N	% Rhythmic	Period (hr) ± S.E.M.	Power^#^ ± S.E.M
*tim-GAL4/+; Pdf-GAL80/+* M *tim-GAL4/+; Pdf-GAL80/* *UAS-miR-210 M* *tim-GAL4/+* M *tim-GAL4/UAS-miR-210* M *Pdf-GAL4/+* M *Pdf-Gal4/UAS-miR-210* M *yw M* *yw F* *miR-210*^*KO*^ *M* *miR-210*^*KO*^ *F* *miR-210*^*KO*^*/+ F* *miR-210*^*KO*^*; UAS-miR-210 M* *Fas2*^*ΔmiR-210*^ *M* *Fas2*^*ΔmiR-210*^ *F* *Fas2*^*ΔmiR-210*^*/+ F* *miR-210*^*KO*^*/Fas2*^*ΔmiR-210*^ *F*	42 35 40 33 41 44 45 42 52 49 31 43 43 39 43 35	100.0±0.0 N/A* 96±2.3 N/A* 96.4±1.4 37.1±3.5 92.3±2.3 91.6±3.6 88.0±2.4 87.5±3.1 90.2±1.9 90.9±2.1 93.2±1.8 94.4±0.8 95.3±1.7 88.6±1.7	24.5±0.4 N/A* 24.1±0.3 N/A* 24.6±0.2 26.1±0.6 24.7±0.2 24.1±0.4 24.2±0.4 24.5±0.3 24.3±0.1 24.6±0.3 24.1±0.2 24.1±0.3 24.3±0.1 23.9±0.4	94.4±2.6 N/A* 97.2±2.5 N/A* 98.4±1.1 65.0±1.5 91.3±1.8 88.7±2.1 87.9±3.2 89.6±1.6 91.4±2.8 86.4±3.1 92.9±2.4 89.8±3.1 93.8±4.2 87.8±2.1

^#^Power is a measure of rhythm amplitude and corresponds to the height of the periodogram peak above the significance line.

*N/A, flies were all dead after entering the constant darkness.

### The molecular pacemaker is intact in the *miR-210*^*KO*^ flies

**Fig 2:**
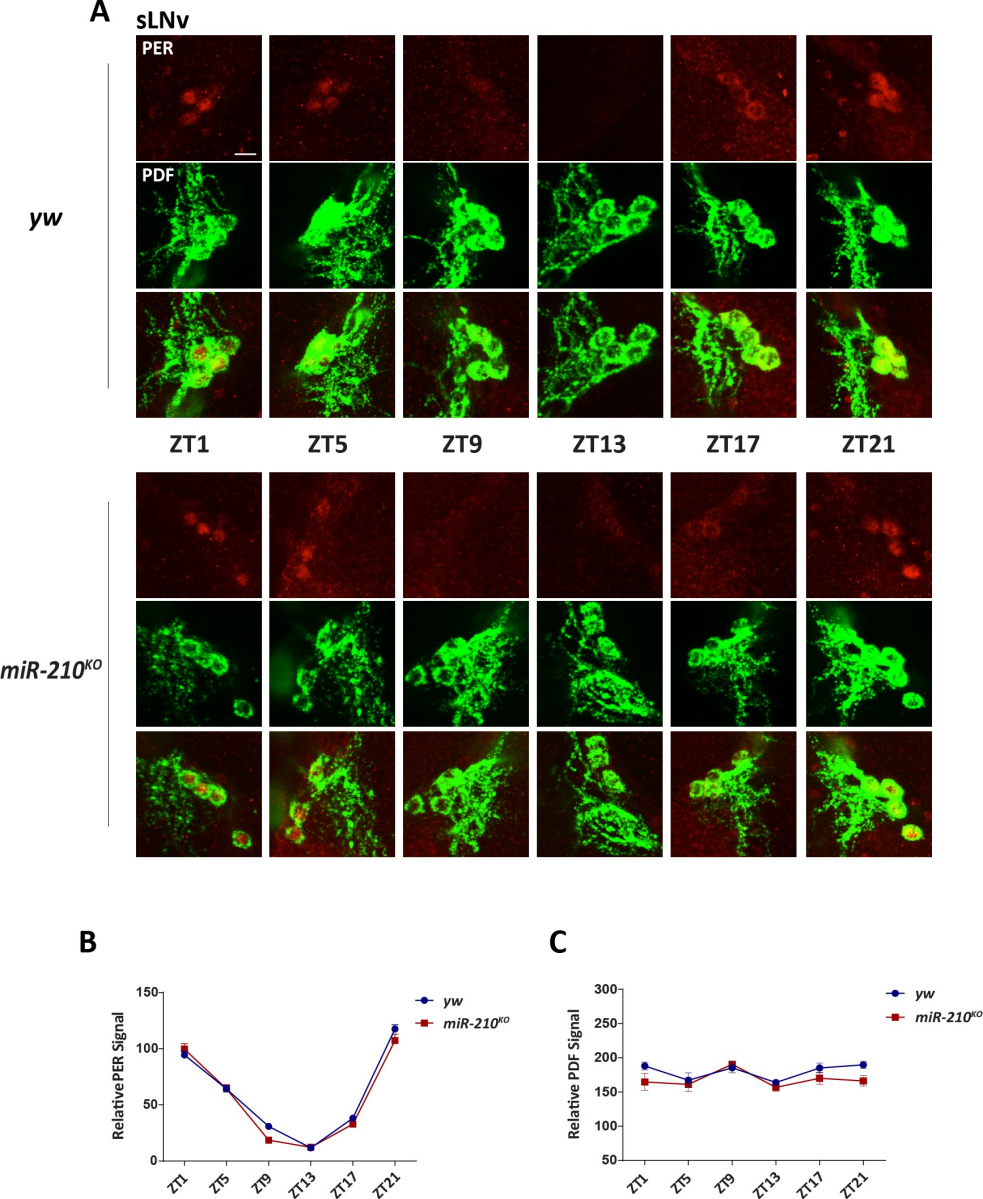
The molecular pacemaker is intact in the *miR-210*^*KO*^ mutants. (A) Representative images of sLNvs in wild-type control and *miR-210*^*KO*^ mutants. Fly brains were dissected at six time points (zeitgeber time, ZT) during the fourth day of LD and stained with anti-PDF (green) and anti-PER (red) antibodies. Scale bar is 10 μm. (B) Quantification of PER staining in sLNv. No significant changes in PER level or cycling were found in *miR-210*^*KO*^. Error bars indicate SEM. (C) Quantification of PDF levels in sLNv. At least twelve brains were quantified. Error bars indicate SEM.

Since the period and rhythmicity of circadian rhythms are unaffected in *miR-210*^*KO*^ flies under DD, it is likely that the molecular pacemaker of *miR-210*^*KO*^ flies is still functional. To confirm this, we examined the oscillation of the key pacemaker protein PER in circadian neurons under DD. We focused on the PDF positive sLNvs, which are the master pacemaker neurons under constant conditions. As expected, we found no obvious changes in PER oscillation or abundance in these sLNvs (S3A and S3B Fig). We further examined PER levels in another two important groups of circadian neurons: LNds, and DN1s. Similar as in sLNvs, PER oscillation in these neurons were not affected either (S3C and S3D Fig). Since we observed the dramatic phase advance phenotype under LD, next we examined the PER oscillation at different times of day. Consistent with the DD results, there was no significant change of PER in the PDF positive sLNvs in *miR-210*^*KO*^ flies (Fig 2A and 2B). Oscillation of PER was unaffected in LNds and DN1s either (S4 Fig). Because PDF is an important circadian output molecule, we also quantified the PDF abundance in sLNvs and found no significant differences in *miR-210*^*KO*^ flies in sLNvs (Fig 2C). Since lLNvs have been identified in regulation of evening activity onset [47], we further examined the PDF abundance of lLNvs across different time of the day. No obvious changes of PDF abundance in lLNv were observed either (S5 Fig). Taken together, these data suggest that loss of *miR-210* has no effects on the molecular clock. The phase advance phenotype is unlikely mediated through changes of PDF abundance.

### Loss of *miR-210* disrupts the circadian arborization rhythms of sLNv dorsal projections

**Fig 3:**
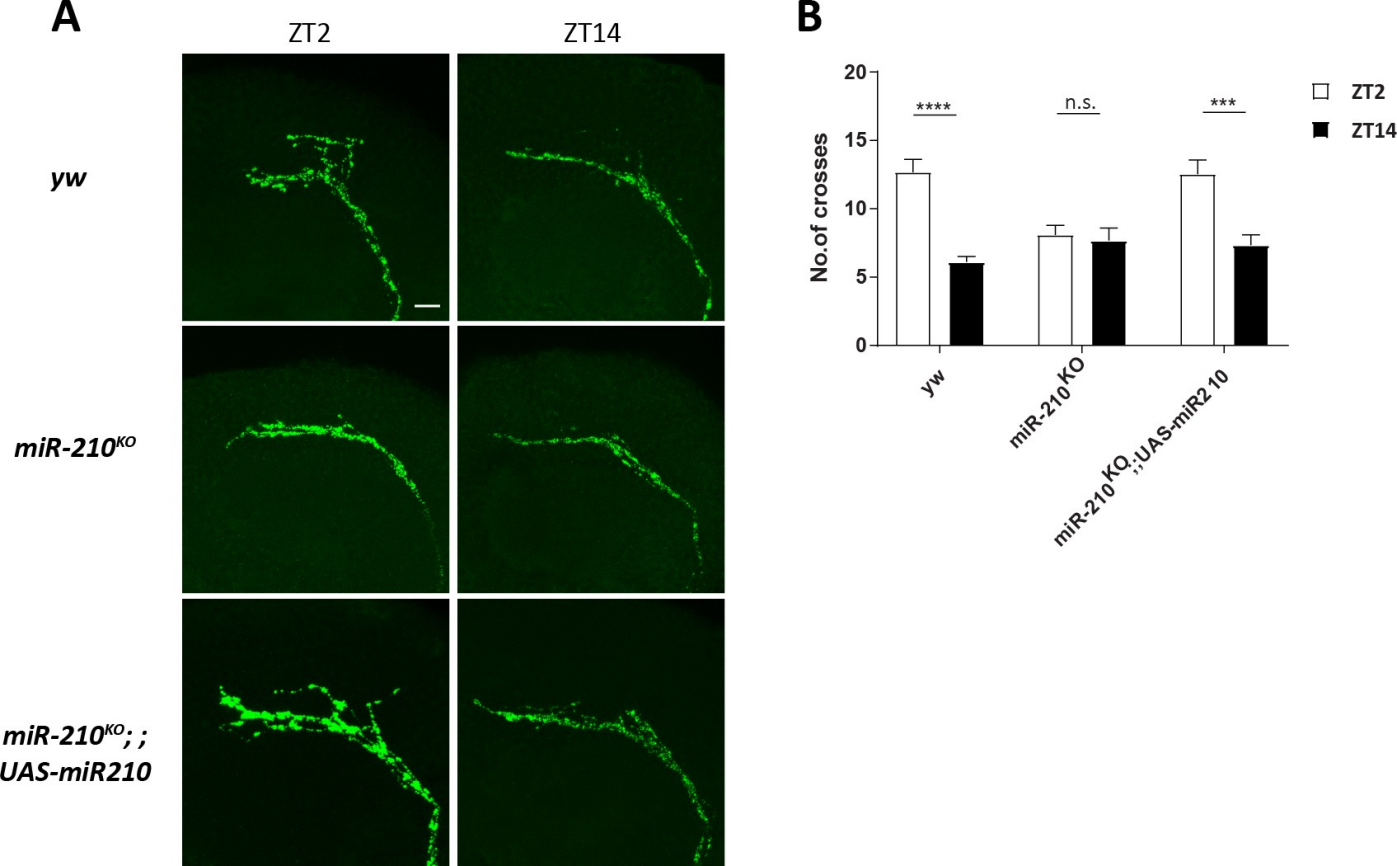
Loss of *miR-210* disrupts the circadian arborization rhythms of sLNv dorsal projections. (A) Representative confocal images of sLNv dorsal axonal projections. The brains were stained with anti-PDF antibody (green) at ZT2 and ZT14 at the 3rd day of LD. Scale bar is 10 μm. (B) Quantification of axonal morphology (fasciculation) of sLNv dorsal termini. Axonal termini defasciculation of sLNv neurons were quantified based on a modified Sholl's analysis. The plot represents number of crosses between axonal branches and concentric rings. Plots show mean values, error bars indicate Standard error. *** represents p-value < 0.001, **** represents < 0.0001 by Student’s t test. ns represents not significant.

Although genetic interaction between *miR-210* and the PDF pathway was identified (S1 Fig), we observed no significant changes in PDF abundance in the flies missing *miR-210* (Fig 2C and S5C Fig). Thus, we decided to examine the PDF projections of the sLNvs. Circadian arborization rhythms of the dorsal projections of sLNvs have been previously observed [14, 15]. To determine whether *miR-210* affects the arborization rhythms of sLNv projections, we used a PDF-specific antibody to examine the termini of sLNv dorsal projections in *miR-210*^*KO*^ flies at early day (Zeitgeber time 2 (ZT2), ZT0 is light on and ZT12 is light off) and early night (ZT14). We found that the wild-type flies had more PDF positive branches of axon terminals at ZT2 than at ZT14 (Fig 3A), as previously observed [15]. Remarkably, this arborization rhythm was abolished in the *miR-210*^*KO*^ flies (Fig 3A). We quantified the dorsal axon arborization using the method as previously described (see Material and Methods) [16] and confirmed that the lack of aborization rhythm in *miR-210*^*KO*^ is mainly due to the significant decrease of axonal crosses at ZT2 compared to control flies (Fig 3B). Furthermore, restoration of *miR-210* expression rescued the arborization rhythm in *miR-210*^*KO*^ mutants (Fig 3A and 3B). Since we found there were no significant changes of PDF abundance in the sLNv soma, these data indicate that the decrease of axonal branches was not due to the decrease of PDF staining (Fig 2 and Fig 3). Together, these results indicate that *miR-210* is required for the circadian arborization rhythms in the sLNv dorsal projections.

### *miR-210* controls the phase of evening peak by targeting Fas2

**Fig 4:**
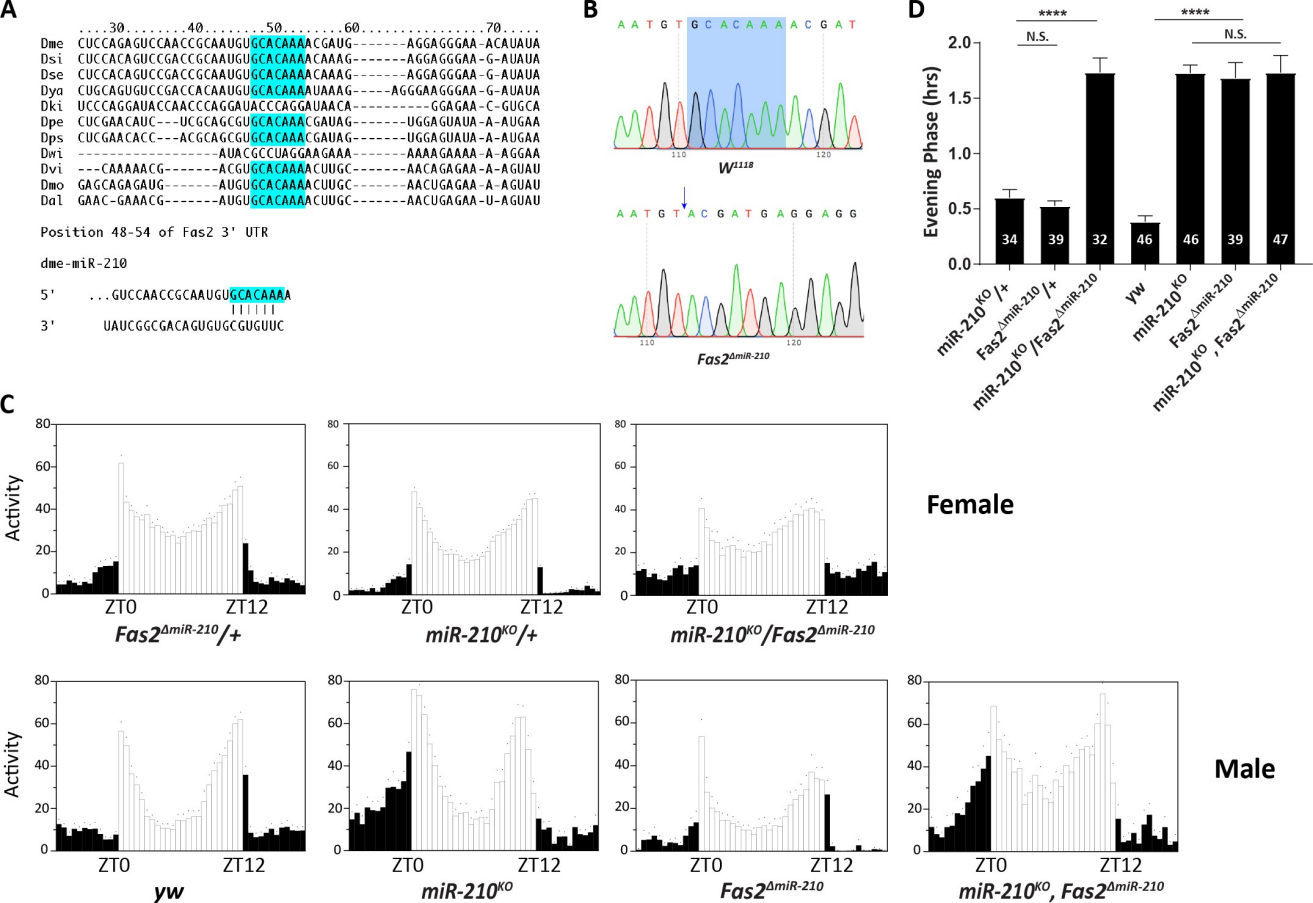
Depletion of putative *miR-210* binding sites within 3’UTR of *Fas2* phenocopies *miR-210*^*KO*^. (A) Predicted *miR-210* binding site in the 3’UTR of *Fas2* is highly conserved across *Drosophila* species. Blue letters indicate conserved sequences matching the 2–7 (seed) sequence of *miR-210*. The *Drosophila* species used for comparison were Dme, *D*. *melanogaster*, Dsi, *D*. *simulans*, Dse, *D*. *secellia*, Dya, *D*. yakuba, Dki, *D*. kikkawai, Dpe, *D*. persimilis, Dps, *D*. pseudoobscura, Dwi, *D*. willistoni, Dvi, *D*. virilis, Dmo, *D*. mojavensis, and Dal, *D*. albomicans. (B) Confirmation of CRISPR/Cas9 mediated deletion of the 7bp from *Fas2* 3’UTR through Sanger sequencing. Blue arrow indicates the place that deletion started. (C) Representative eduction profiles of locomotor activity under 12:12 LD conditions. Since both *miR-210* and *Fas2* are located on the X chromosome, females were used in the upper panel for genetic interactions in transheterozygous. Lower panel shows male controls and *miR-210*^*KO*^, *Fas2*^*ΔmiR-210*^ double mutant flies. (D) Quantification of phase of evening peak. Error bars indicate Standard Error. **** = p<0.0001, determined by Student’s t test. ns represents not significant.

miRNAs play negative posttranscriptional functions through binding to the 3’ untranslated regions (UTRs) of target mRNAs [31]. After searching putative *miR-210* targets by using an *in silico* prediction algorithm (http://www.targetscan.org/fly_12/), we identified *Fas*2 as one of the potential targets (Fig 4A). The putative *miR-210* binding sites within the 3’UTR of *Fas2* are highly conserved across most *Drosophila* species (Fig 4A). Interestingly, Sivachenko et al. have reported that overexpression of *Fas2* in the PDF neurons caused fasciculation of sLNv dorsal projections and disrupted arborization rhythms [15]. Since miRNAs are normally negative regulators of target genes, and overexpression of *Fas2* recapitulated the anatomical phenotype of *miR-210*^*KO*^ flies, we decided to use the CRISPR-Cas9 system to delete the *miR-210* binding site within the *Fas2* 3’UTR. As the seed region of miRNAs (positions 2–7) is critical for miRNA function [48], to minimize potential off-target effects, we managed to delete only 7 bps of the matching sequence on the 3’UTR (Fig 4B). We recovered several lines, which were homozygous viable, and named the mutant as *Fas2*^*ΔmiR-210*^.

Next we tested the circadian locomotor behavior of the *Fas2*^*ΔmiR-210*^. There was no change in period or rhythmicity under DD (Table 1). However, the *Fas2*^*ΔmiR-210*^ flies clearly advanced the phase of evening activity, similar to *miR-210*^*KO*^ flies (Fig 4C and 4D). To determine whether *Fas2* and *miR-210* function in the same pathway, we tested the potential genetic interactions using two methods. First, we tested the *miR-210*^*KO*^/ *Fas2*^*ΔmiR-210*^ transheterzygous flies. While both *miR-210*^*KO*^ and *Fas2*^*ΔmiR-210*^ are recessive mutations, the transheterzygous flies had a strikingly similar phase advance phenotype as single mutants (Fig 4C and 4D). Second, we examined the double mutant of *miR-210*^*KO*^, *Fas2*^*ΔmiR-210*^ flies. Under LD, the *miR-210*^*KO*^, *Fas2*^*ΔmiR-210*^ flies exhibited a similar phase advance phenotype, which is insignificant from either *miR-210*^*KO*^ or *Fas2*^*ΔmiR-210*^ (Fig 4C and 4D). This data indicated that *miR-210* and *Fas2* function in the same pathway regulating circadian phase of activity. Taken together, *Fas2* genetically interacts with *miR-210*, and ablation of the *miR-210* binding sites within the 3’UTR of *Fas2* recapitulated the behavior phenotypes of *miR-210*^*KO*^ mutants. These results indicate that *Fas2* is a functional target of *miR-210*.

### *miR-210* regulates circadian rhythms through inhibition of Fas2 in the optic lobe

**Fig 5:**
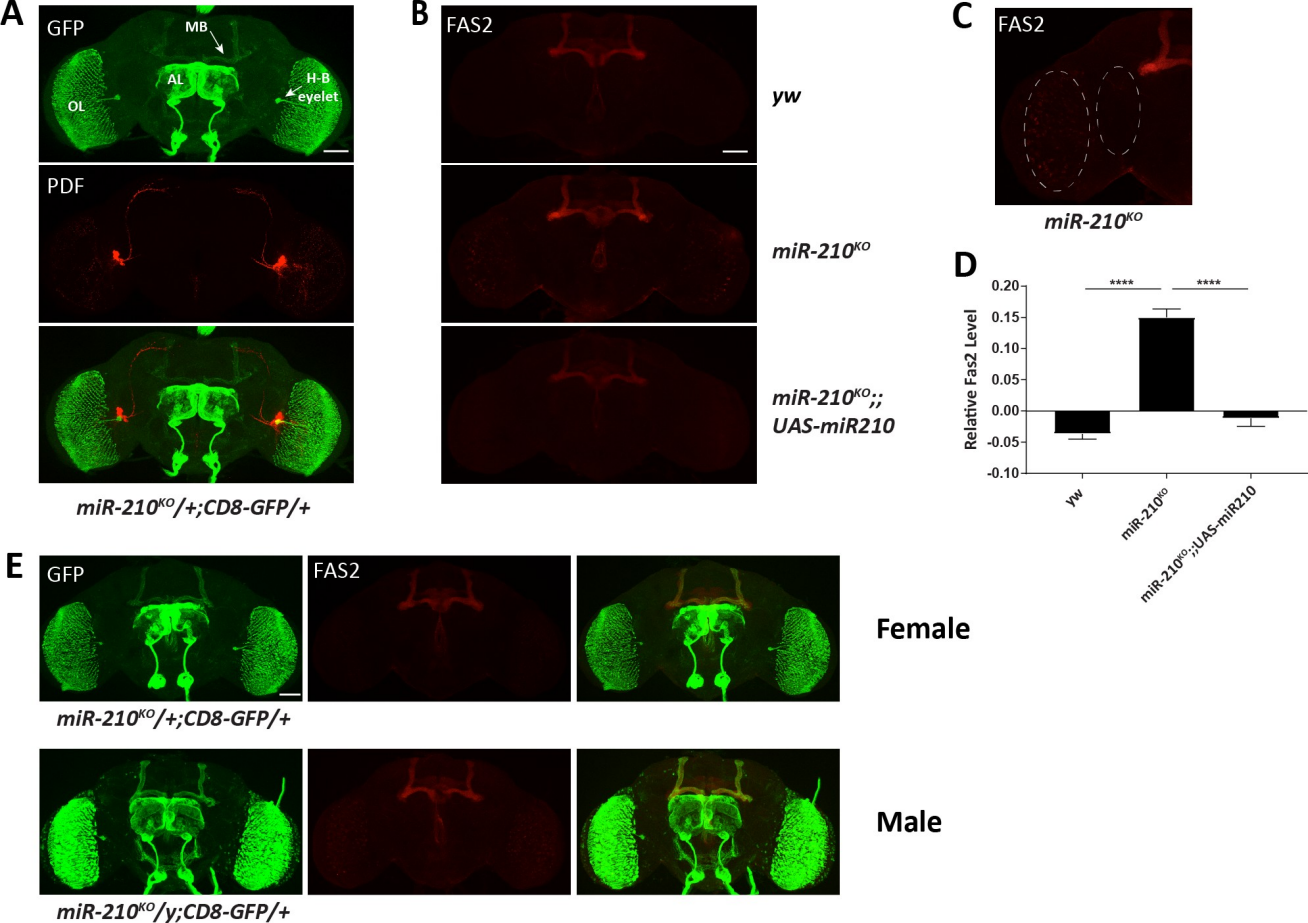
Fas2 abundance is elevated in the optic lobe of *miR-210*^*KO*^ flies. (A) Representative image showing *miR-210* expression profile in fly brain. Green signal represents *miR-210* expression (driven by *miR-210*^*KO*^ Gal4), while red indicates PDF. OL, AL, H-B eyelet, and MB represents optic lobe, antennal lobe, Hofbauer-Buchner eyelet, and mushroom bodies, respectively. White arrows indicates the MB and H-B eyelet. (B) Representative confocal images for Fas2 staining in fly brains. Note that Fas2 abundance was elevated in the optic lobes of *miR-210*^*KO*^ and normal in rescue flies. Scale bar is 100 μm in A-B. (C) Schematic representing area luminance analysis for quantification of relative Fas2 level in the optic lobe. Quantification is based on measure of the Fas2 signal in the optic lobe (same size, dash lines) and subtraction of brain region next to it (small dash line circle). (D) Quantification of Fas2 in the optic lobe per square μm. Error bars indicate Standard Error. **** = p<0.0001, determined by Student’s t test. (E) Loss of *miR-210* increases Fas2 levels in the cells where *miR-210* was expressed. Fly brains stained with anti-GFP (green) and Fas2 (red). Upper panel is female heterozygous flies expressing CD8-GFP. Lower panel shows the Fas2 expression increased in the optic lobes of male mutant flies. Scale bar is 100 μm.

To identify the cellular mechanism of *miR-210* functions, we first characterized the *miR-210* expression pattern in the fly brain by using the *miR-210* knock-in Gal4 to drive GFP as a reporter. We observed GFP signals in the optic lobe, Hofbauer-Buchner eyelet (H-B eyelet), as well as several other brain regions, including the mushroom bodies and antennal lobe (Fig 5A). To our surprise, no GFP signal was detected in LNvs as labeled by anti-PDF antibody staining. Then we examined Fas2 expression using the commercial antibody (1D4) from DSHB. As previously reported, in wild type flies Fas2 was strongly expressed in the mushroom bodies as well as the ellipsoid bodies (Fig 5B) [49]. Interestingly, we detected an intensive Fas2 signal in the optic lobe of *miR-210*^*KO*^ mutant flies, which disappeared when *miR-210* expression was rescued (Fig 5B). We quantified Fas2 abundance in the optic lobe and observed significant increase of Fas2 in *miR-210*^*KO*^ mutants compared to the wild-type and rescue (Fig 5C and 5D). Lastly, we wondered whether the increase of Fas2 abundance occurs at the same sites where *miR-210* is expressed. While female heterozygous *miR-210*^*KO*^ flies exhibited no detectable Fas2 in the optic lobe, dramatic increase of Fas2 signal clearly overlapped with *miR-210* expression in the optic lobe (Fig 4E).

**Fig 6:**
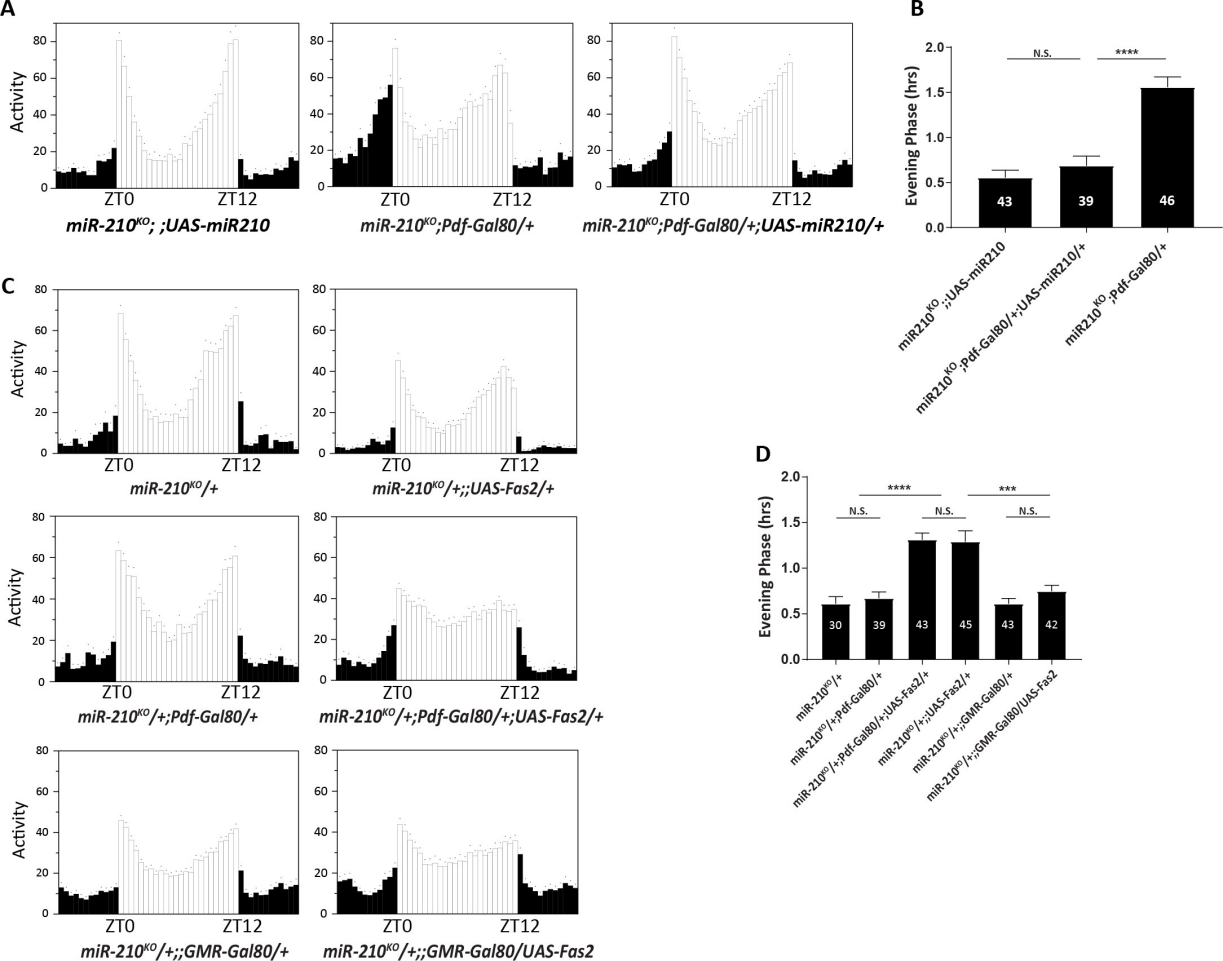
*miR-210* is required in the optic lobe for controlling the phase of evening behavior. (A) Representative eduction profiles of flies under 12:12 LD cycle. *miR-210* expression in PDF negative cells is sufficient to rescue the phase advance in evening peaks. (B) Quantification of evening phase in A. (C) Overexpression of *Fas2* in *miR-210* expressing non-photoreceptor cells did not advance the phase of evening peaks. Upper panel shows overexpression of *Fas2* in *miR-210* cells advanced the evening phase. Middle panel shows blocking *Fas2* expression in the PDF cells still advanced the phase. Female flies were used in this figure C. (D) Quantification of evening phase in C. Error bars indicate Standard Error. ns represents not significant. *** = p<0.001, **** = p<0.0001 was determined by Student’s t test.

Although we did not detect *miR-210* expression in circadian neurons with the *miR-210* knock-in Gal4, the single cell RT-PCR data from Chen and Rosbash indicated that *miR-210* has an oscillating expression in the sLNvs [50]. Thus, we first tested the requirement of *miR-210* in the sLNvs for normal circadian behavioral rhythms under LD, combining *miR-210*^*KO*^ GAL4 and *Pdf*-GAL80. GAL80 is a repressor of GAL4 function, and *Pdf*-GAL80 has been efficiently used to inhibit gene expression in the PDF neurons [7, 51]. Surprisingly, we observed a clear rescue of evening phase when we excluded *miR-210* expression in the sLNvs, which suggests that *miR-210* is not necessary in PDF neurons for circadian behavior (Fig 6A and 6B). Next, we tested whether overexpression of *Fas2* in the *miR-210-*expression cells would mimic the behavior phenotype of *miR-210*^*KO*^ mutants using the *miR-210*^*KO*^ GAL4. While heterozygous *miR-210*^*KO*^ mutant has no phase advance, we observed a weaker (~1.5 hr) but significant advance of evening peak in *Fas2* overexpression (Fig 6C and 6D). Consistent with the *miR-210* data, Fas2 is not required in the PDF positive neurons (Fig 6C and 6D). Since increase of Fas2 in the optic lobe was observed in the *miR-210*^*KO*^ mutants, we tested whether up-regulation of Fas2 is responsible for the phase advance phenotype. When we used GMR-GAL80 to block Fas2 expression in the photoreceptors, the advance of evening phase was rescued (Fig 6C and 6D). Together these data suggest that inhibition of Fas2 by *miR-210* in the optic lobe is critical for the timing of evening activity.

## Discussion

Emerging roles of miRNA in the control of different aspects of circadian rhythms have been recently uncovered [33]. Here, we screen *Drosophila* miRNA mutants and demonstrate that *miR-210* is critical for circadian locomotor rhythms. *miR-210* determines the proper phase of evening activity peak under entrainment. *miR-210* plays its role in circadian rhythms and axonal arborization via repression of *Fas2*.

*Drosophila* gradually increases its locomotor behavior and reaches its peak of evening activity at light off. The molecular mechanism underlying the phase control of evening peak is still largely unknown. Loss of *miR-210* advanced the evening phase to the same extent as *pdf*^*01*^ mutants. Beside PDF, other neuropeptides such as ITP and NPF also play roles in regulation of evening peak. Downregulation of ITP in circadian neurons has weak effects on evening phase and also prolongs circadian period under constant darkness [52]. While ablation of NPF positive circadian neurons advances the phasing of evening activity, it also affects period during free running [53]. These phenotypes are inconsistent with what we observed in *miR-210*^*KO*^ mutants (Table 1). In fact, our genetic interaction data suggests that *miR-210* functions in the same signaling pathway as PDF. So we decided to focus on PDF pathway. However, unlike the *pdf*^*01*^ mutants [11], loss of *miR-210* has little effect on period and circadian rhythmicity. These results indicate that *miR-210* may specifically regulate one aspect of PDF signaling functions.

Circadian structural plasticity of sLNv dorsal projections has been recently identified [54, 55]. The biological function and molecular mechanisms underlying the structural plasticity however remain elusive. Here we identified that *miR-210*^*KO*^ mutants disrupted the axonal arborization rhythms of sLNv, which is likely through inhibition of the neural cell adhesion molecule Fas2. Deletion of the 7-bp seed region of *miR-210* binding sites within the 3’UTR of *Fas2* mimics the effect of *miR-210*^*KO*^ mutants on the sLNv arborization rhythm. However it seems unlikely that the phase advanced phenotype of *miR-210*^*KO*^ mutants is because of the defects in sLNv arborization rhythms. First, as far as we know, previous genetic manipulations that cause severe defects on sLNv structural plasticity have no effect on evening phase [54, 55]. Second, although overexpression or down regulation of Fas2 in the sLNv abolished the arborization rhythms, it does not affect the evening phase either [16]. So we conclude that these effects of *miR-210* on evening phase control and axonal structural plasticity may not be functionally related.

Our evidence that *Fas2* is a key target for *miR-210* in regulation of circadian rhythms is particularly strong. First, *miR-210*^*KO*^ mutants elevated the Fas2 abundance in the optic lobe, where *miR-210* is highly expressed. This is consistent with the general role of miRNAs as negative regulators of target proteins. Second, *Fas2*^*ΔmiR-210*^ recapitulates the arborization and circadian behavior phenotypes of loss of *miR-210*. Furthermore, our genetic interaction data also indicates that *Fas2* and *miR-210* function in the same pathway.

In which cells are *miR-210* and *Fas2* required for the evening phase of activity? Transcriptome profiling from the Rosbash lab show that expressions of both *miR-210* and *Fas2* are enriched and oscillating in the PDF positive neurons [50]. Overexpressing *miR-210* in the PDF neurons causes flies to become arrhythmic or rhythmic with a prolonged circadian period (Table 1). However, using *miR-210* knock-in GAL4 or Fas2 antibodies, we were not able to detect obvious signals for *miR-210* or Fas2 in the PDF neurons. We cannot exclude the possibility that there is weak amount of *miR-210* or Fas2 protein expression, which is not detectable due to sensitivity of the techniques we used. We sought to answer this question by utilizing the well-used *Pdf*-GAL80 repressor. Indeed, restoring *miR-210* in its endogenous expressing cells except PDF neurons rescued the circadian behavior completely. This data indicates that expression of *miR-210* in the PDF neurons is not necessary for its circadian function. Consistent with this notion, overexpression of *Fas2* in PDF negative but *miR-210* expressing cells causes a phase advance of evening peak. Since the *miR-210* knock-in driver has no detectable expression in the PDF neurons, leaky expression due to potential weak suppression of *Pdf*-GAL80 should not be an alternative explanation. The H-B eyelets interact with lLNvs and control the phase of evening peak activity [47, 56]. Interestingly, *miR-210* exhibits strong signals of expression in the H-B eyelets (Fig 5A). We labeled H-B eyelets in *miR-210*^*KO*^ or *Fas2*^*ΔmiR-210*^ by expressing GFP in the Rh6-Gal4 (S6 Fig). In wild-type flies, H-B eyelet axons projected close to the LNvs as previously described [47]. We did not observe obvious defects of H-B eyelet in these two mutants (S6 Fig). However, we cannot exclude the possibility that *miR-210*^*KO*^ or *Fas2*^*ΔmiR-210*^ mutants disrupt the communication between H-B eyelets and lLNv, thus affect the evening activity.

Our data suggest that inhibition of Fas2 by *miR-210* in the optic lobe is critical for the proper evening phase. How does miR-210-Fas2 expression in the optic lobe control the phase? One possibility is that Fas2 expression in the optic lobe may affect the phase through PDF signaling in the lLNv. If that’s the case, Fas2 abundance in the optic lobe may show differences at different time points. Thus we examined the Fas2 abundance across different time of the day in the optic lobe (S7A Fig). High amount of Fas2 was observed in the *miR-210*^*KO*^ compared to the wild-type, however, no oscillation was identified in either genotype (S7B Fig). This result is consistent with the fact that we did not observe changes of PDF in lLNv (S6 Fig). So it is unlikely that miR-210-Fas2 affect the evening phase through changes of PDF level in the ILNv. However, we cannot exclude the possibility that it may modulate PDF-receptor signaling or other pathways. It is worth to mention that during the preparation of this paper, another independent study also characterizes the role of *miR-210* in phase control of circadian behavior and reached similar conclusion with us on the circadian behavior and dorsal aborization phenotype [57]. Even though no specific target was functionally validated, it is possible that some of the pathways identified in that paper may also contribute to the phase control phenotypes in the *miR-210*^*KO*^.

Here we identify that *miR-210* regulates circadian locomotor rhythms and axonal structural plasticity of pacemaker neurons in *Drosophila*. A similar mechanism may exist in mammals. Interestingly, the glutamatergic synapses on the VIP neurons of mammalian master circadian pacemaker suprachiasmatic nucleus (SCN) also show circadian structural plasticity, which maybe important for light entrainment in mice. *miR-210* is a highly conserved miRNA from worms, flies, to humans. *In silico* miRNA target prediction with targetscan identified a highly conserved binding site of human *miR-210* in the 3’UTR of vertebrates *brain-derived neurotrophic factor* (BDNF*)*. BDNF has well-established functions in axonal branching and synaptic plasticity [58, 59]. Thus, it is worthwhile to test that whether *miR-210* plays conserved functions in structural plasticity in mammals.

## Material and methods

### (A) Fly stocks

All the flies were raised on standard cornmeal/agar medium at 25°C under 12:12 hour LD cycle. The following strains were used in this study: *w*^*1118*^, *yw*, *pdf*^*01*^, *y w; tim-GAL4/CyO* [46], *PDF-Gal80*, *y w; Pdf-GAL4/CyO* [7], *UAS-miR-210*, *UAS-Fas2*, *miR-210*^*KO*^ [44], *UAS-CD8-GFP* (BL5137), *GMR-Gal80* [60], *Rh6-GAL4* (BL7464), *Fas2*^*ΔmiR-210*^ *(generated by CRISPR-Cas9 in our lab)*.

### Behavioral experiments and analysis

Most of the times, adult male flies (2–5 days old) were used to test locomotor activity rhythms. Since both *miR-210* and *Fas2* are both on the x chromosome, adult female flies were also used for locomotor rhythms, which were mentioned in the relevant figures. Flies were entrained for 4 days LD cycle at 25°C and 60% humidity, and released into constant darkness (DD) at 25°C for at least 5 days. Temperature cycles were performed with 12h:12h 29C:21C cycling for 6d in complete darkness. Trikinetics *Drosophila* activity monitors were used to record locomotor activity in I36-LL Percival incubators. Activity rhythms were analyzed with Faasx software protocol [61]. Eduction profiles were generated with 3d of LD activity. FAAS-X software was used to analyze behavioral data [60]. Actograms were generated with a signal-processing toolbox for MATLAB. The evening anticipation amplitude was determined by assaying for the locomotor activity as described.

### Immunohistochemistry and quantification

Immunohistochemistry was performed with whole *Drosophila* brains. For staining involving ZT timepoints, Flies were entrained to LD for 3 d and dissected at Zeitgeber time (ZT) 0, 2, or 14. For CT staining, flies were entrained to LD, released to DD, and dissected on the second day of DD at 6 CT intervals. Mouse anti-Fas2 (1:100), mouse anti-PDF (1:400), Rabbit anti-GFP 1:200, Rabbit anti-PER (1:1500) was used. All brains were imaged with a leica confocal microscope. Microscope laser settings were held constant for each experiment. ImageJ was used for quantification of PER, PDF and FAS 2 luminance. At least 5 brains were used for quantification. ImageJ was used in the analysis of axonal morphology (fasciculation) of sLNv dorsal termini by a modified Sholl's analysis [56].

### Design of CRISPR mediated deletion and screening of Fas2 3’UTR deletions

*Fas2*^*ΔmiR-210*^ flies were generated by CRISPR mediated deletion by Rainbow Transgenic Flies. The guide RNA target sequence for CRISPR was GCAATGTGCACAAAACGATGAGG. The primers used for genomic *miR-210* f3: GCCAACAGGCAGCATCAAAC, and *miR-210* r3: CCAACTTAGTGTGCCAATCGATC. PCR amplification of the *Fas2* 3’ UTR region containing the *miR-210* motif was performed by using *miR-210* f3 and *miR-210* r3 primers. Sequencing of the PCR products was performed by using the *miR-210* f3 primer. Identification of homozygous *Fas2*^*ΔmiR-210*^ flies was confirmed by observing a 7bp deletion at the *miR-210* binding motif in the sequence chromatograph.

## Supporting information

S1 Fig Loss of *miR-210* advances the evening phase under constant darkness. (A) Locomoter behavior profile in DD. Time of circadian peak activity is shown on the graph. The number 0 and 12 shows CT (circadian time). Gray indicates the subjective night. (B) Quantification of the time of peak activity in DD. * = p<0.05, **** = p<0.0001, determined by Student’s t test.
(PDF)

S2 Fig *miR-210* genetically interacts with *pdf*. (A) Representative eduction profiles of fly locomotor activity under 12:12 LD cycle. Black represents the dark phase, while white represents the light phase. Eduction is analyzed based on average of 3 days LD. (B) Quantification of evening phase of flies under LD. Number of flies tested is listed in each bar. (C) Representative eduction profiles under 16:8 LD cycle. (D) Quantification of evening phase of flies under 16:8 LD. Number of flies tested is listed in each bar. Error bars indicate Standard Error. **** = p<0.0001, determined by Student’s t test.
(PDF)

S3 Fig The molecular pacemaker is intact in the *miR-210^KO^* mutants under DD. (A) Representative images of sLNvs in wild-type control and *miR-210^KO^* mutants. Fly brains were dissected at six time points (circadian time, CT) during the second day of DD and stained with anti-PDF (green) and anti-PER (red) antibodies. Scale bar is 10 μm. (B) Quantification of PER staining in sLNv. No significant changes in PER level or cycling. Error bars indicate SEM. (C) Quantification of PER levels in DN1. (D) Quantification of PER levels in LNd. Error bars indicate SEM.
(PDF)

S4 Fig PER oscillation is not affected in the DN1 and LNds of *miR-210^KO^* mutants under LD. (A-B) Representative images of DN1 (A) and LNd (B). Fly brains were dissected and stained same as Fig 2. Scale bar is 10 μm. (C-D) Quantification of PER staining in DN1 (C) and LNd (D). Error bars indicate SEM. ns = no significance, determined by Student’s t test.
(PDF)

S5 Fig PDF abundance is not affected in the lLNv of *miR-210^KO^* mutants under LD. (A) Representative images of lLNv in wild-type and *miR-210*^*KO*^ mutant fly brain. Flies were dissected at six time points on the 4^th^ day of LD and brains were stained same as Fig 2. Scale bar is 20 μm. (B) Quantification of PER staining in ILNv. (C) Quantification of PDF staining in ILNv. At least 12 brains were quantified.
(PDF)

S6 Fig No obvious defect in the H-B eyelet structure is detected in the *miR-210^KO^* or *Fas2^ΔmiR-210^* mutants. Upper panel shows the whole brain staining of PDF (red) and GFP (green) in wild-type, *miR-210*^*KO*^, and *Fas2*^*ΔmiR-210*^ mutants. Lower panel shows the enlarged image of the optic lobe and LNv region. Rh6-Gal4 is used here as a marker for the H-B eyelet. Six pointed white stars indicate the H-B eyelet terminal in fly brain.
(PDF)

S7 Fig No oscillation of Fas2 abundance is detected in the *miR-210^KO^* mutants. (A) Representative images of fly brains at different time points under LD. Flies were entrained under LD cycles and brains were dissected at the 4^th^ day in LD. Red, Fas2 staining, green, GFP. Rh6-Gal4 was used to label R8 positive photoreceptors and H-B eyelet. Note the increase of Fas2 is found in the optic lobe of *miR-210*^*KO*^ at all six time points. Scale bar is 10 μm. (B) Quantification of Fas2 in the optic lobe of wild-type and *miR-210*^*KO*^. Fas2 is constantly higher in the *miR-210*^*KO*^ mutant but does not show an oscillation. Fas2 abundance is quantified as Fig 5C.
(PDF)

S1 Table Locomotor activity of miR[KO] flis in DD. The flybase number of the lines used here are: 58880 (mir-10), 58885 (mir-1007), 58888 (mir-1014), 58889 (mir-1017), 58890 (mir-11), 58892 (mir-133), 58894 (mir-13b-2), 58896 (mir-184), 58898 (mir-193), 58899 (mir-210), 58900 (mir-219), 58904 (mir-274), 58908 (mir-277-34), 58909 (mir-278), 58910 (mir-281-1-281-2), 58911 (mir-282), 58912 (mir-283), 58913 (mir-284), 58914 (mir-285), 58915 (mir-2b-1), 58916 (mir-2c-13a-1), 58917 (mir-303), 58924 (mir-314), 58926 (mir-317), 58928 (mir-31a), 58929 (mir-31b), 58930 (mir-33), 58892 (mir-133), 58935 (mir-927), 58937 (mir-92a), 58939 (mir-932), 58940 (mir-955), 58942 (mir-957), 58943 (mir-958), 58948 (mir-967), 58949 (mir-968-1002), 58952 (mir-971), 58953 (mir-971-972-973), 58954 (mir-975-976-977), 58959 (mir-986), 58960 (mir-987), 58961 (mir-988), 58962 (mir-989), 58963 (mir-990), 58966 (mir-999), 58960 (mir-987), 58967 (mir-9c).
(DOCX)

S2 Table Locomotor activity of *miR-210^KO^* flies in TC. (DOCX)
