# Supplementary figures and images for: miR-210 controls the evening phase of circadian locomotor rhythms through repression of Fasciclin 2
Source: PLoS Genet. 2019 Jul 29;15(7):e1007655. doi: 10.1371/journal.pgen.1007655 (PMC6687186; doi:10.1371/journal.pgen.1007655)

**A**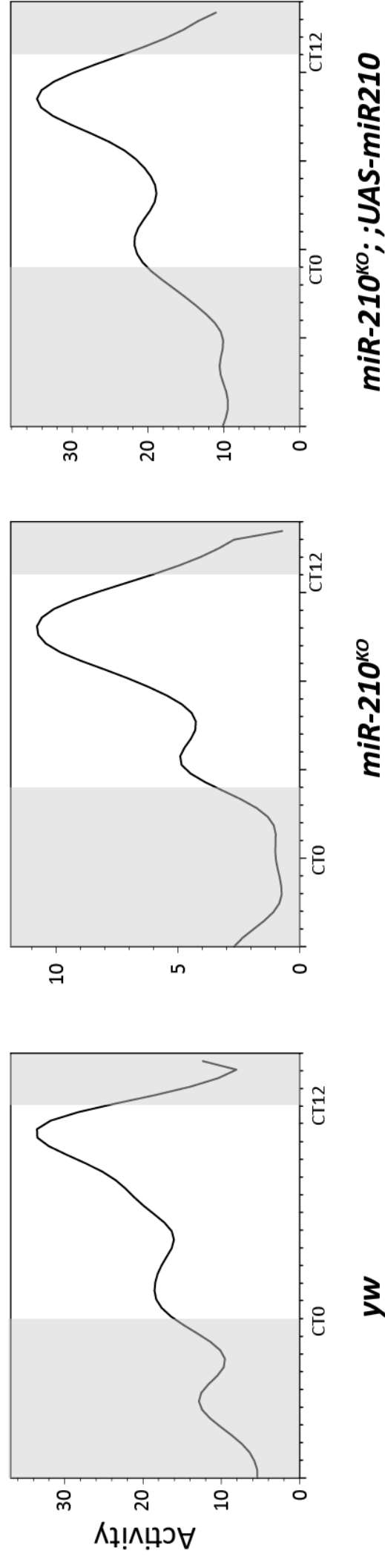**B**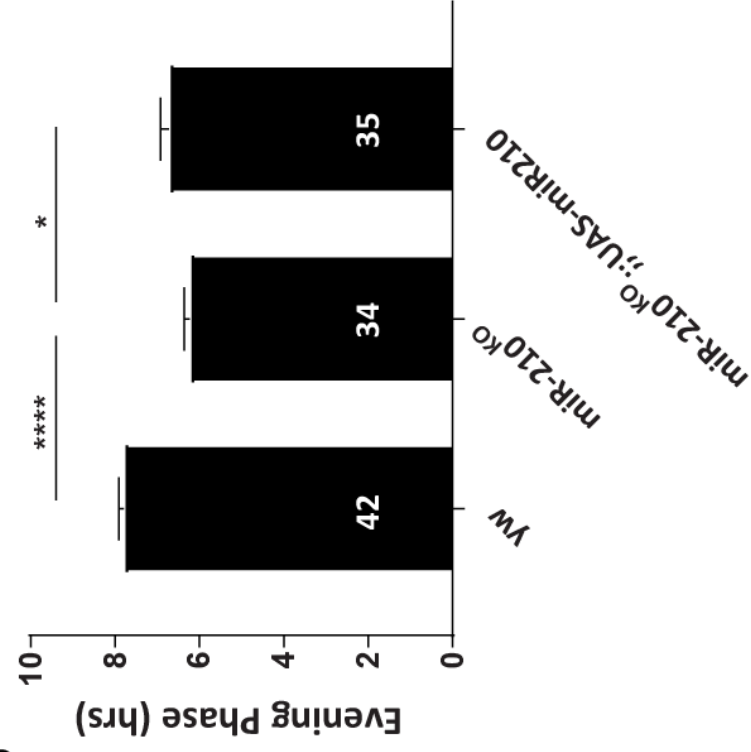

Supplement: S1 Fig — (A) Locomoter behavior profile in DD. Time of circadian peak activity is shown on the graph. The number 0 and 12 shows CT (circadian time). Gray indicates the subjective night. (B) Quantification of the time of peak activity in DD. * = p<0.05, **** = p<0.0001, determined by Student’s t test. (PDF) [file pgen.1007655.s001.pdf]

A

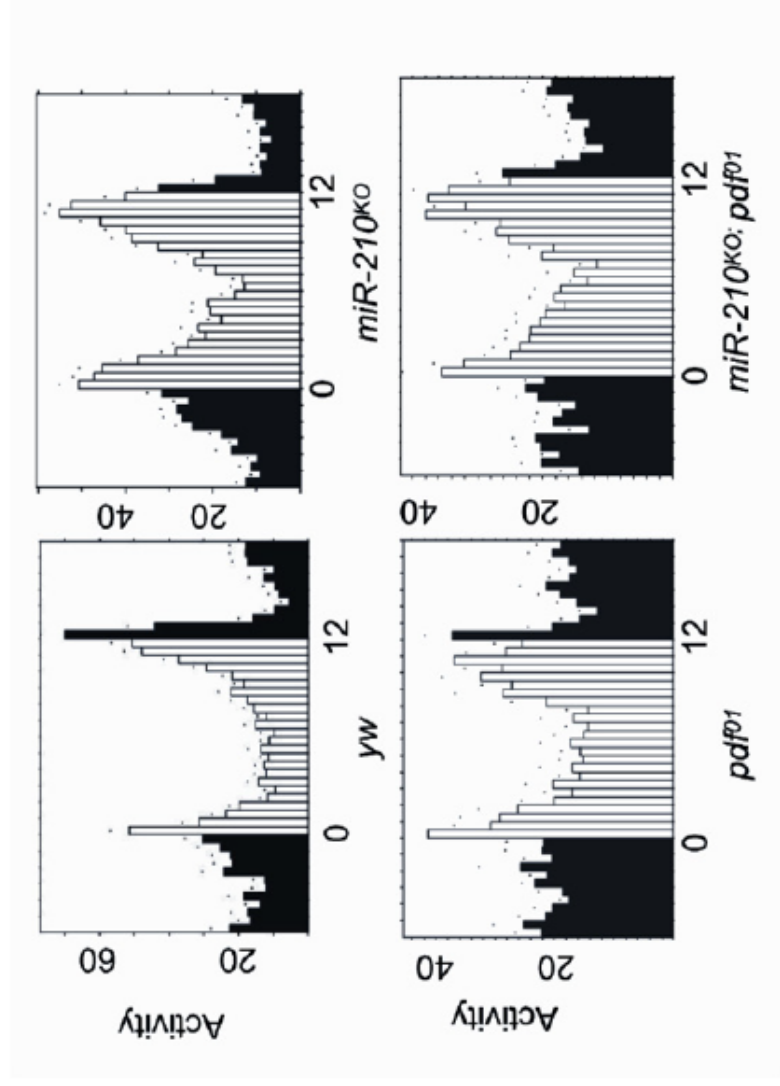

C

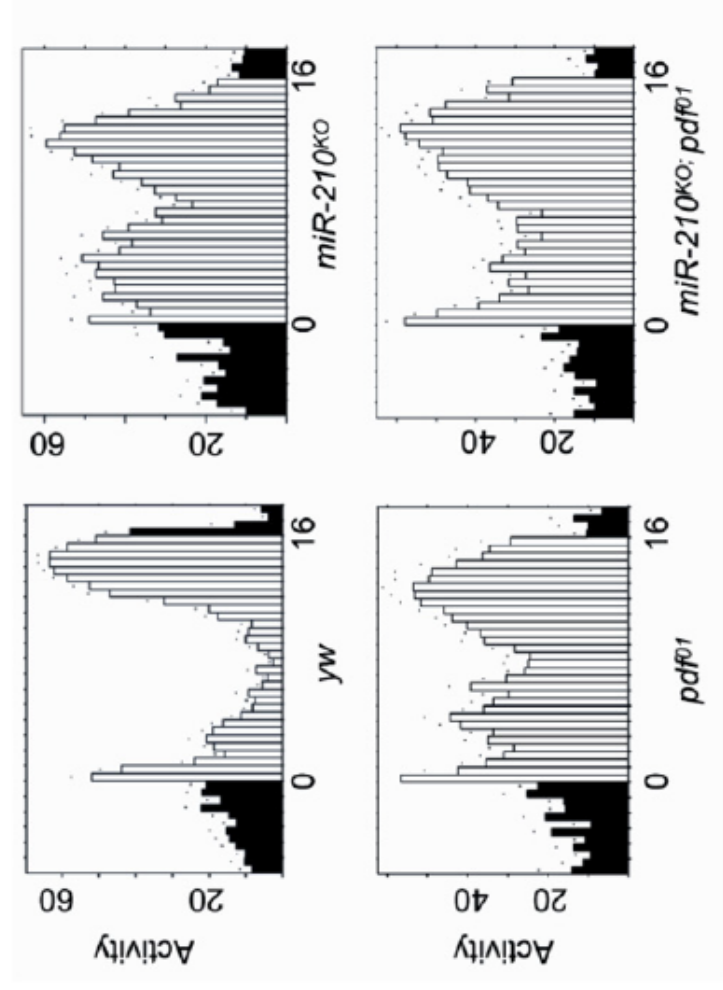

B

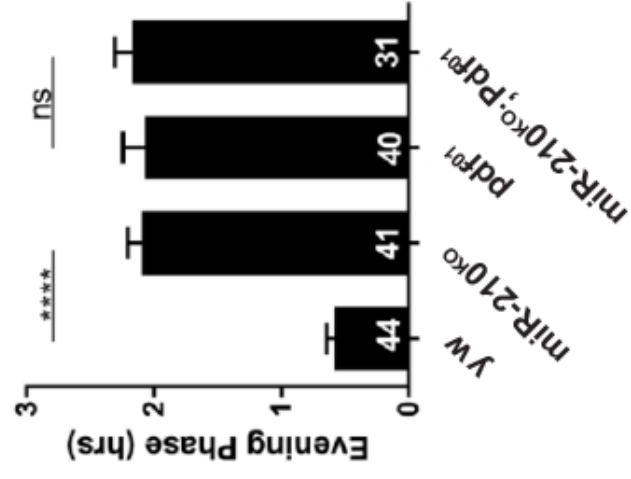

D

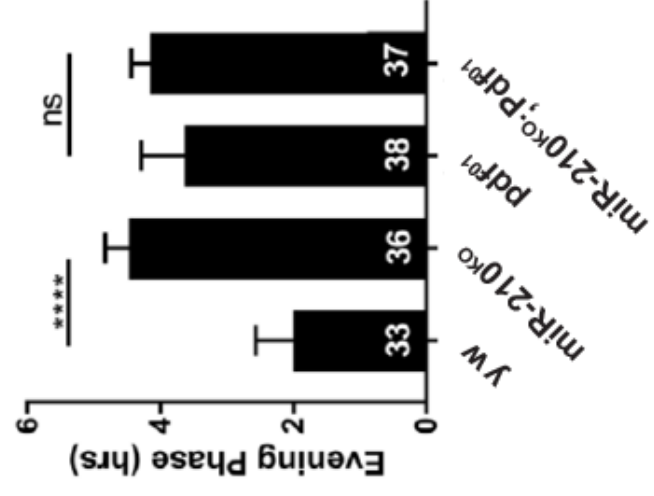

Supplement: S2 Fig — (A) Representative eduction profiles of fly locomotor activity under 12:12 LD cycle. Black represents the dark phase, while white represents the light phase. Eduction is analyzed based on average of 3 days LD. (B) Quantification of evening phase of flies under LD. Number of flies tested is listed in each bar. (C) Representative eduction profiles under 16:8 LD cycle. (D) Quantification of evening phase of flies under 16:8 LD. Number of flies tested is listed in each bar. Error bars indicate Standard Error. **** = p<0.0001, determined by Student’s t test. (PDF) [file pgen.1007655.s002.pdf]

**A**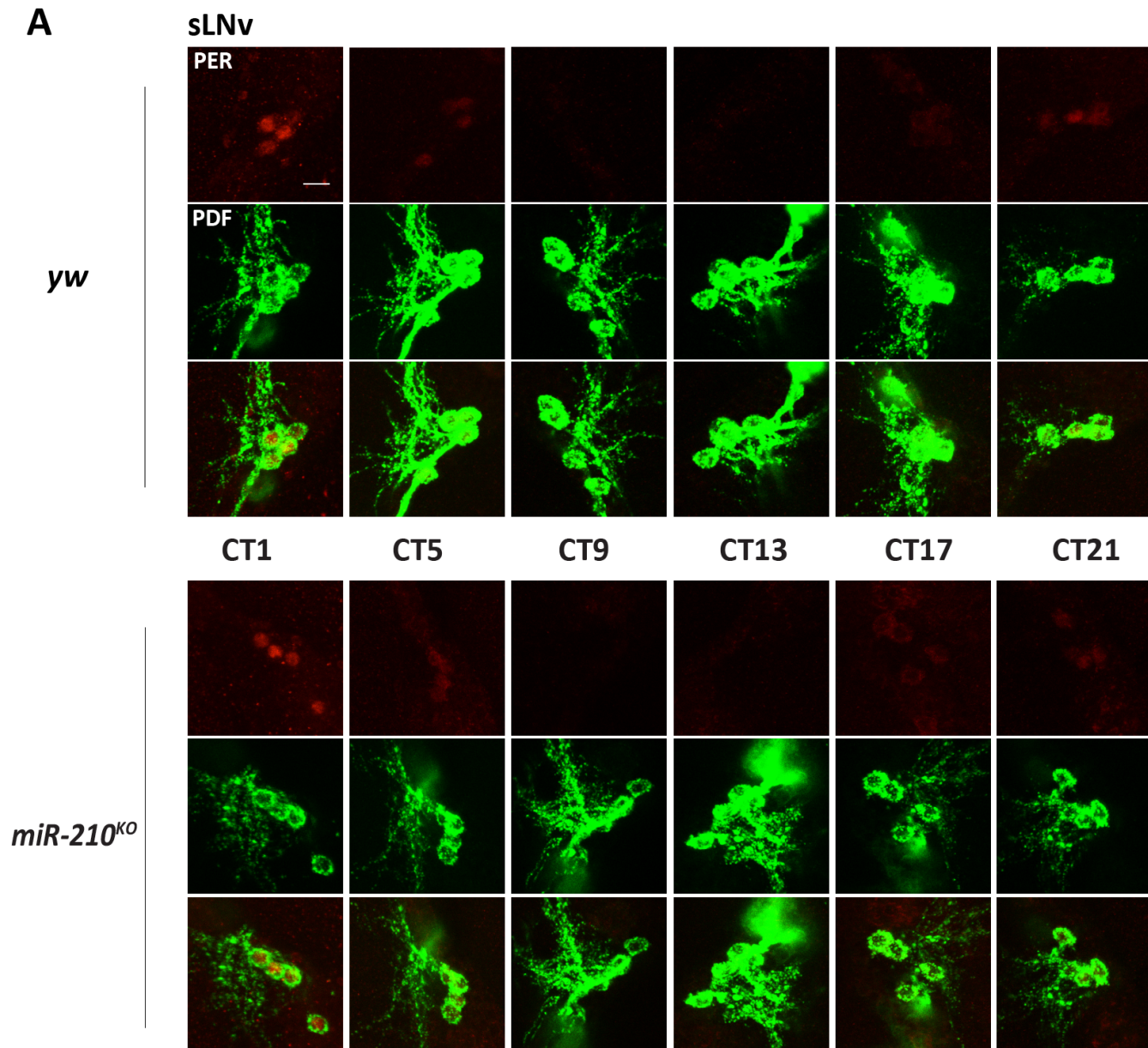**B**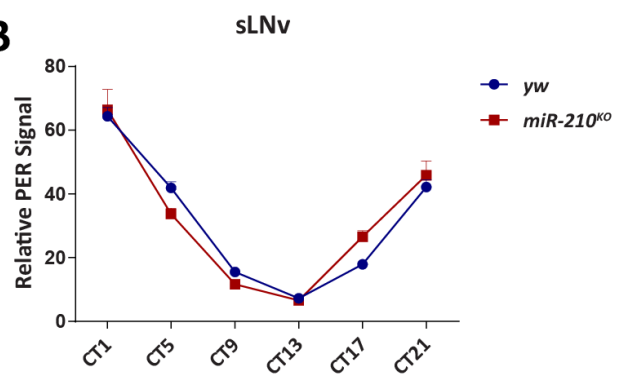**C**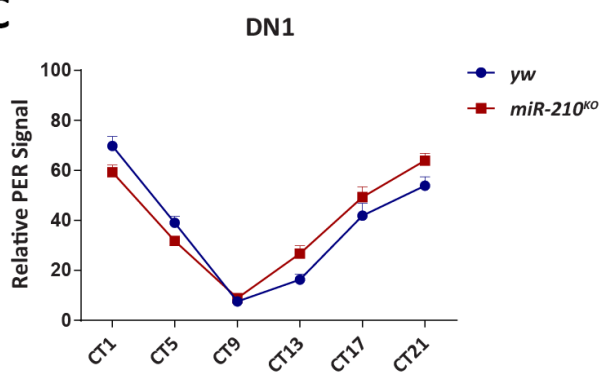**D**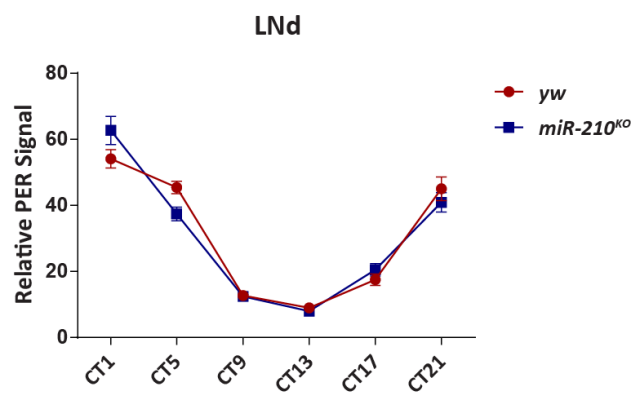

Supplement: S3 Fig — (A) Representative images of sLNvs in wild-type control and miR-210KO mutants. Fly brains were dissected at six time points (circadian time, CT) during the second day of DD and stained with anti-PDF (green) and anti-PER (red) antibodies. Scale bar is 10 μm. (B) Quantification of PER staining in sLNv. No significant changes in PER level or cycling. Error bars indicate SEM. (C) Quantification of PER levels in DN1. (D) Quantification of PER levels in LNd. Error bars indicate SEM. (PDF) [file pgen.1007655.s003.pdf]

**A**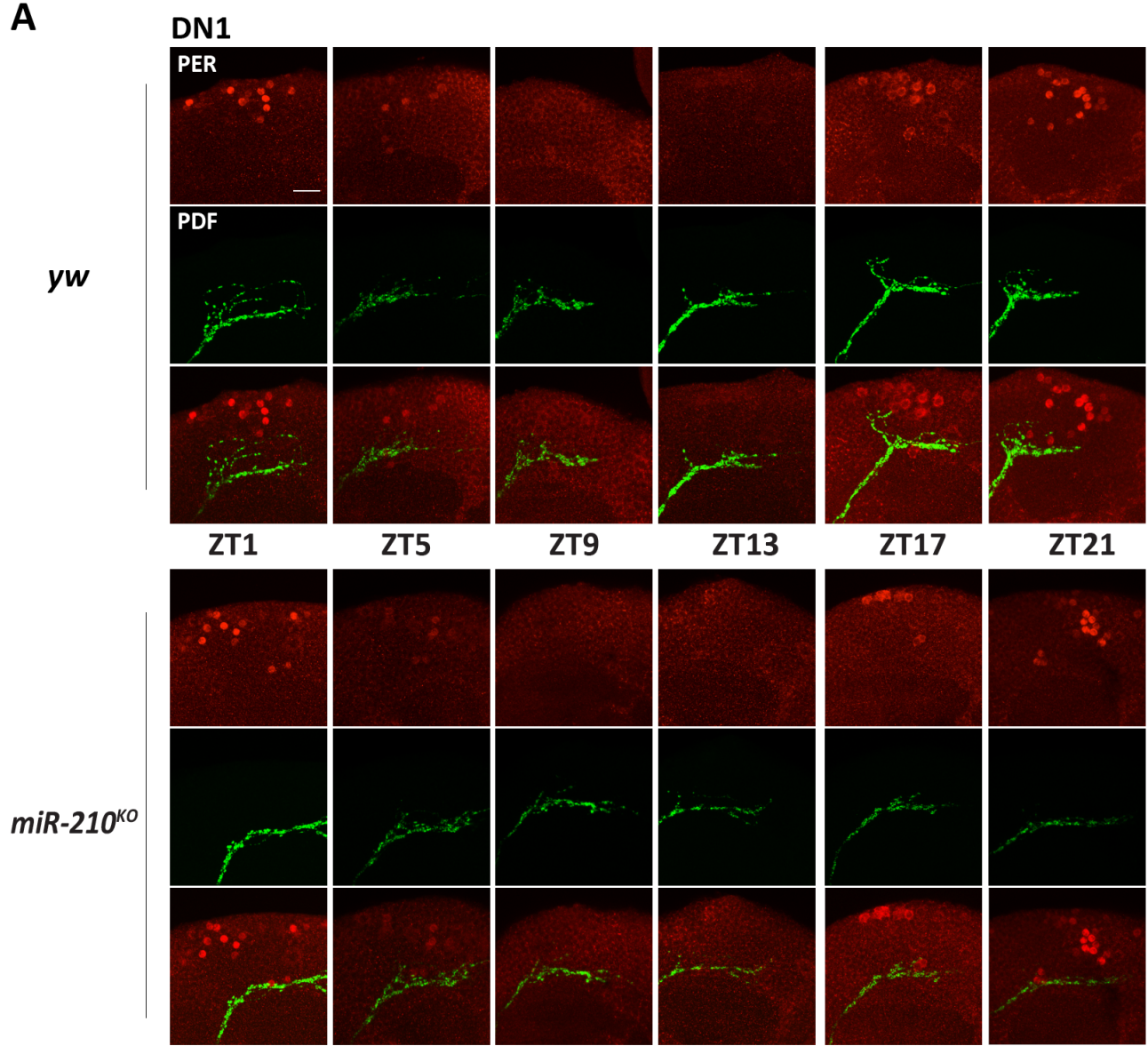**B**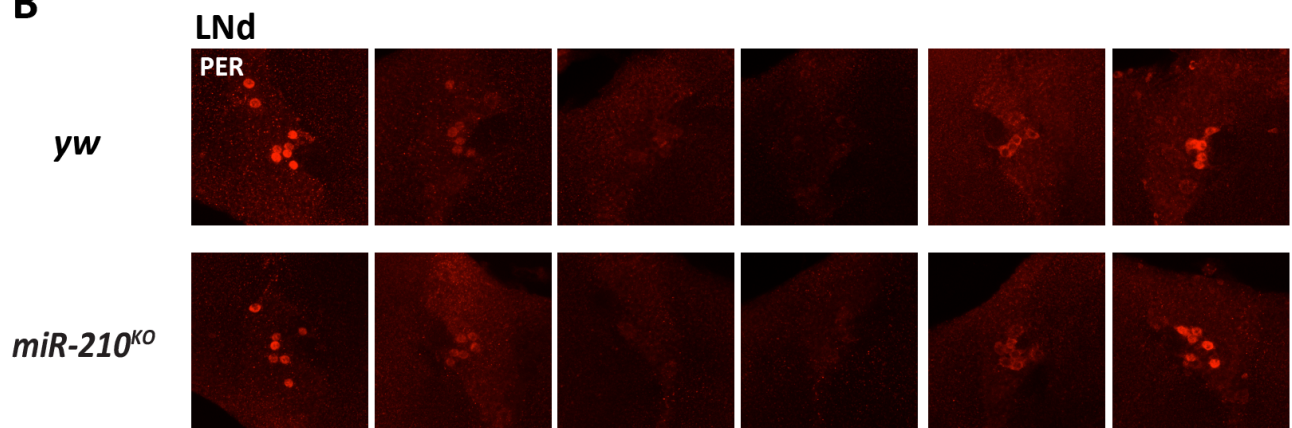**C**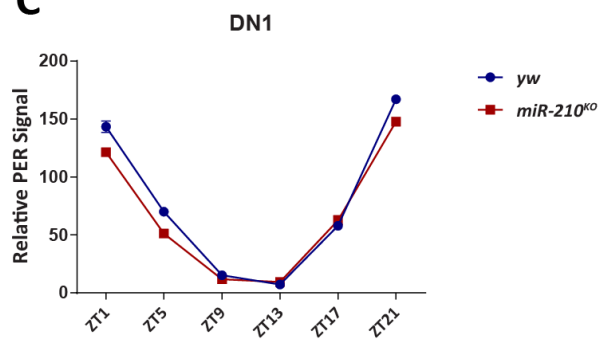**D**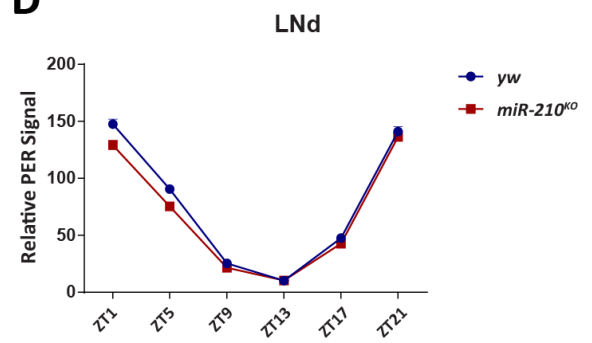

Supplement: S4 Fig — (A-B) Representative images of DN1 (A) and LNd (B). Fly brains were dissected and stained same as Fig 2. Scale bar is 10 μm. (C-D) Quantification of PER staining in DN1 (C) and LNd (D). Error bars indicate SEM. ns = no significance, determined by Student’s t test. (PDF) [file pgen.1007655.s004.pdf]

**A**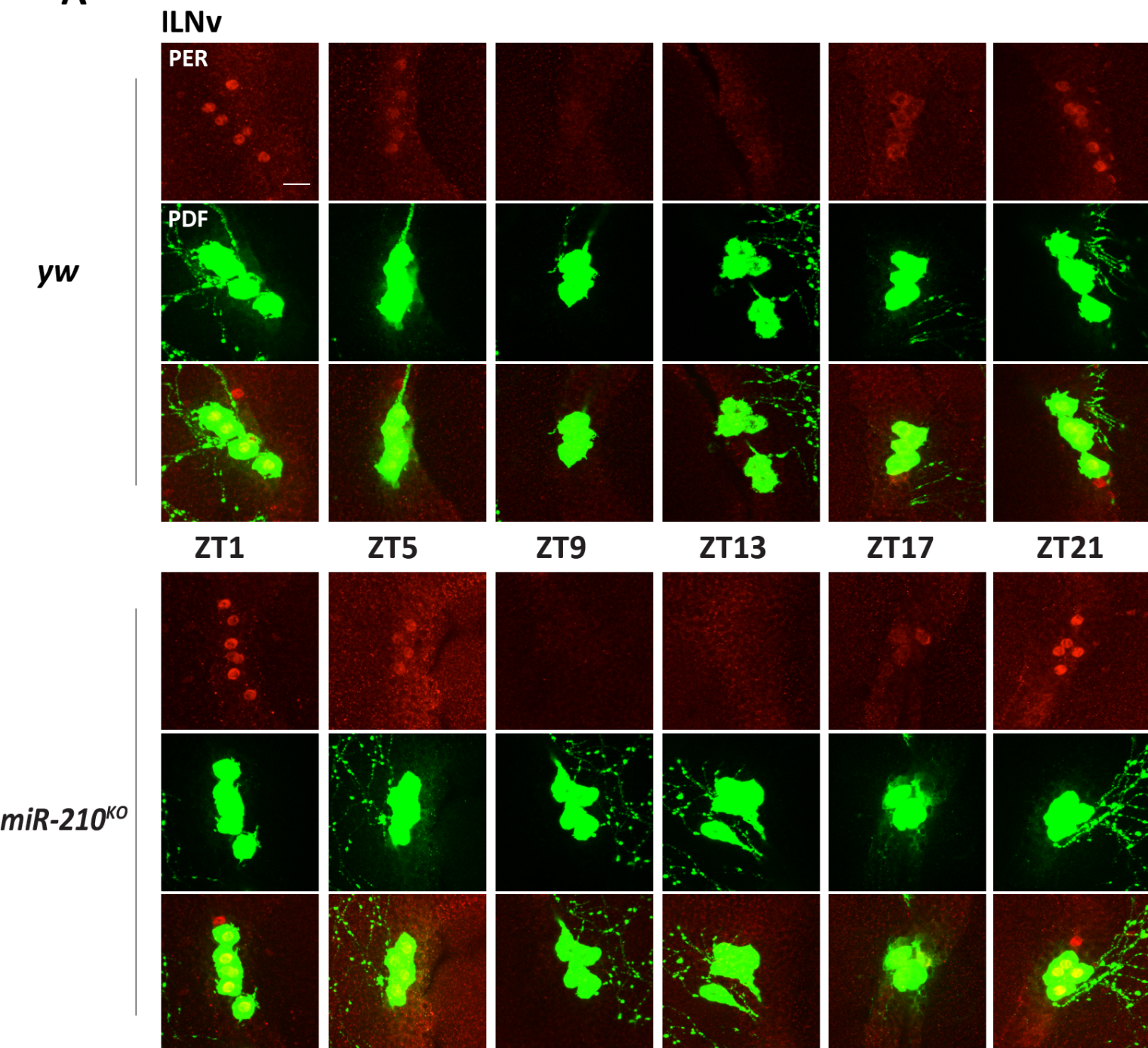**B**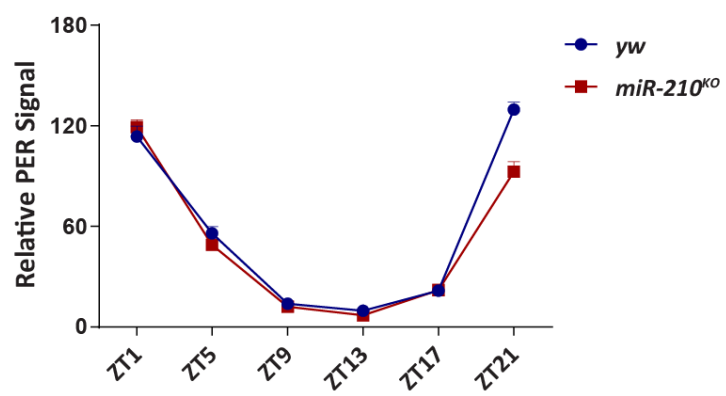**C**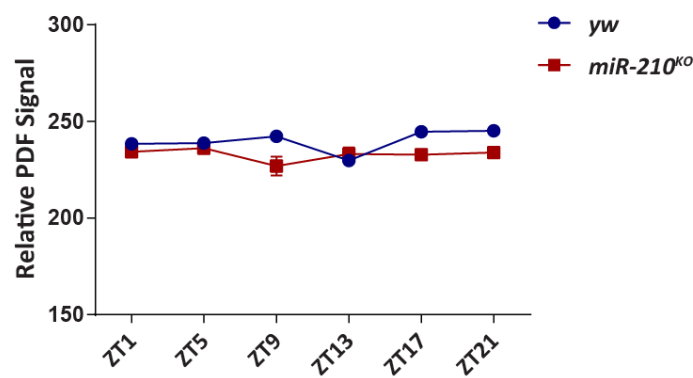

Supplement: S5 Fig — (A) Representative images of lLNv in wild-type and miR-210KO mutant fly brain. Flies were dissected at six time points on the 4th day of LD and brains were stained same as Fig 2. Scale bar is 20 μm. (B) Quantification of PER staining in ILNv. (C) Quantification of PDF staining in ILNv. At least 12 brains were quantified. (PDF) [file pgen.1007655.s005.pdf]

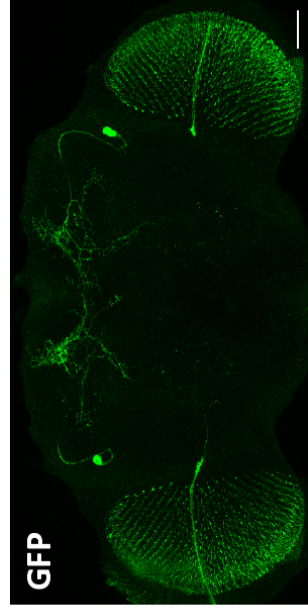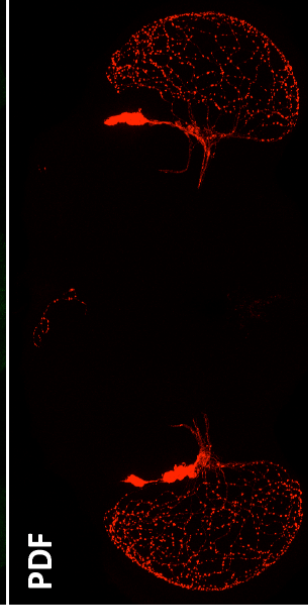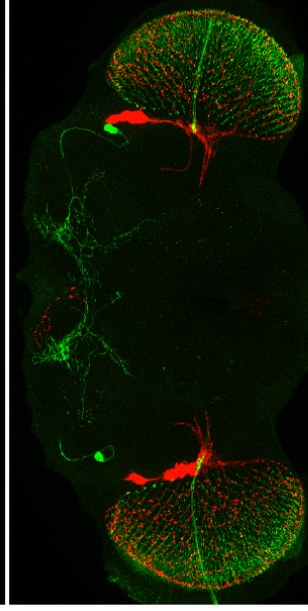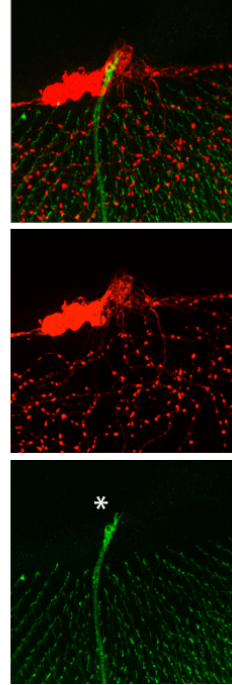

*Rh6-Gal4/+;CD8-GFP/+*

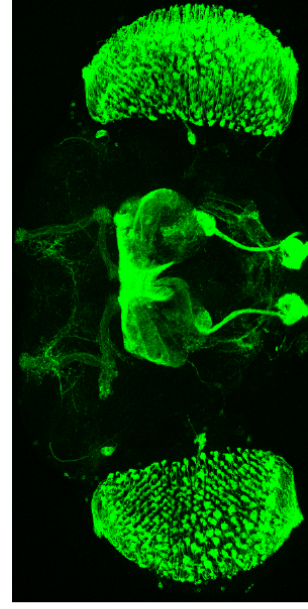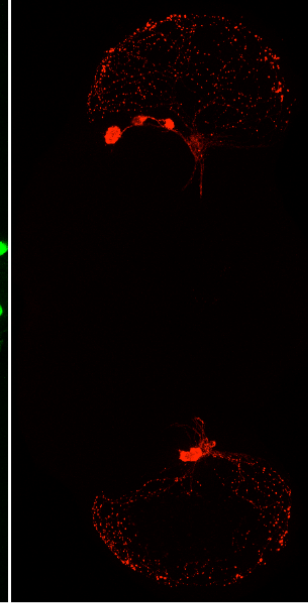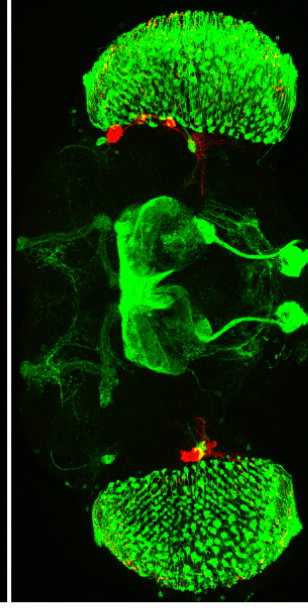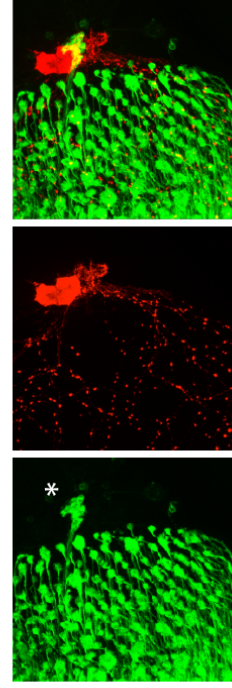

*miR-210<sup>KO</sup>;Rh6-Gal4/+;CD8-GFP/+*

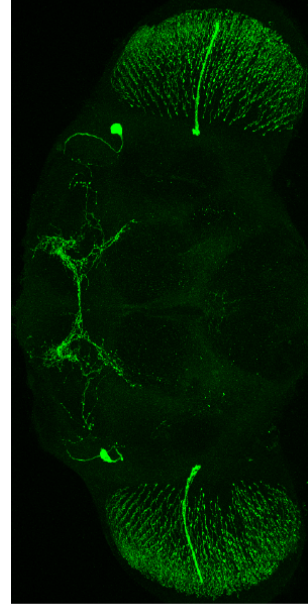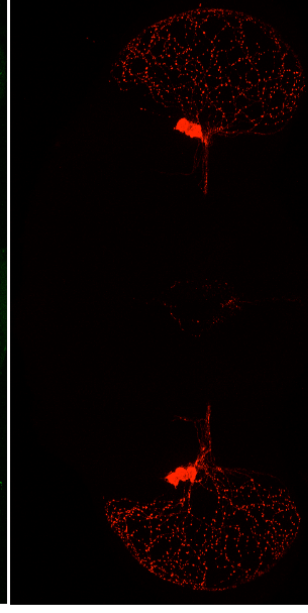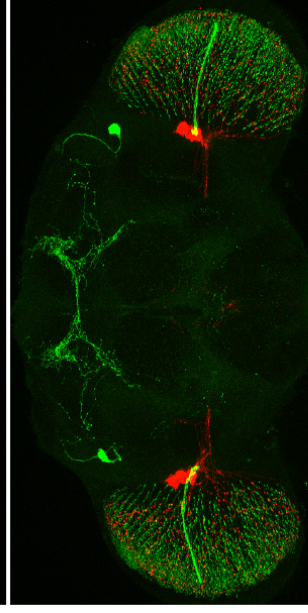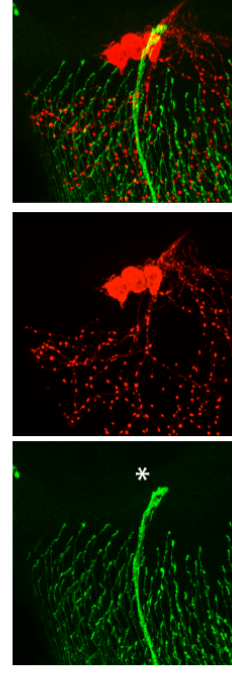

*Fas2<sup>ΔmiR-210</sup>;Rh6-Gal4/+;CD8-GFP/+*

Supplement: S6 Fig — Upper panel shows the whole brain staining of PDF (red) and GFP (green) in wild-type, miR-210KO, and Fas2ΔmiR-210 mutants. Lower panel shows the enlarged image of the optic lobe and LNv region. Rh6-Gal4 is used here as a marker for the H-B eyelet. Six pointed white stars indicate the H-B eyelet terminal in fly brain. (PDF) [file pgen.1007655.s006.pdf]

**A***miR-210<sup>KO</sup>;Rh6-Gal4/+;CD8-GFP/+**Rh6-Gal4/+;CD8-GFP/+*

ZT1

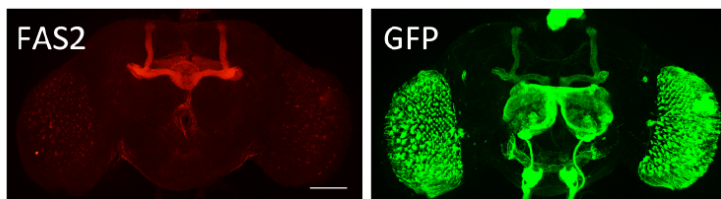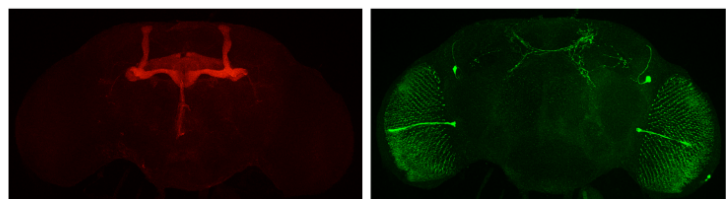

ZT5

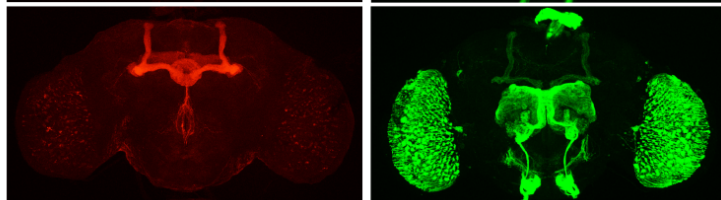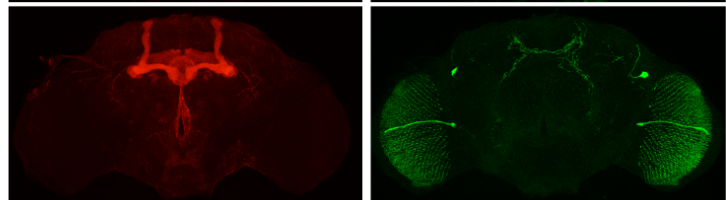

ZT9

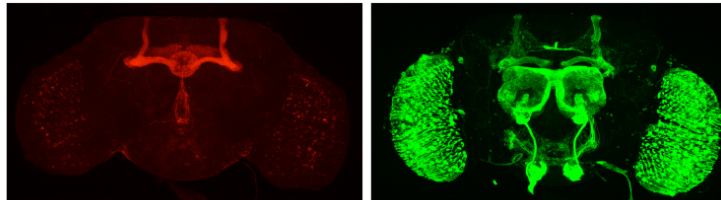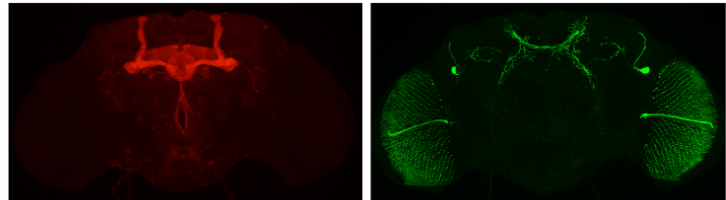

ZT13

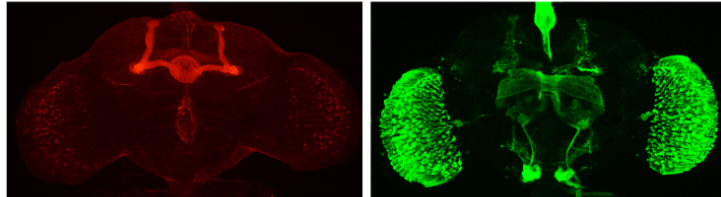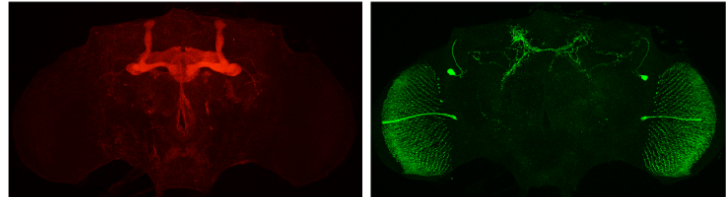

ZT17

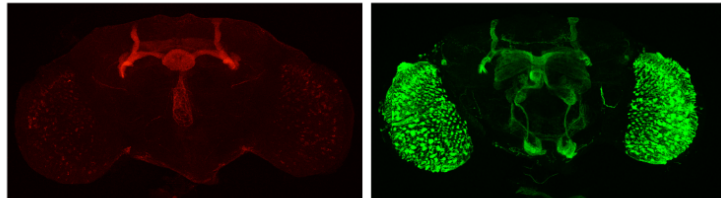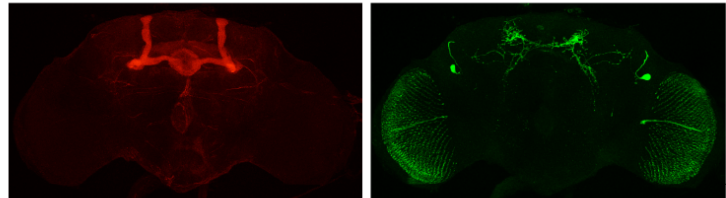

ZT21

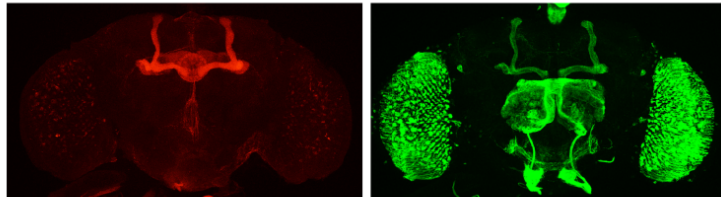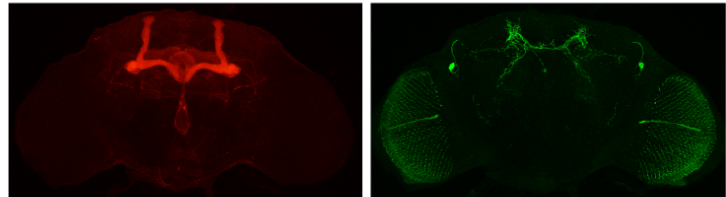**B**

optic lobe

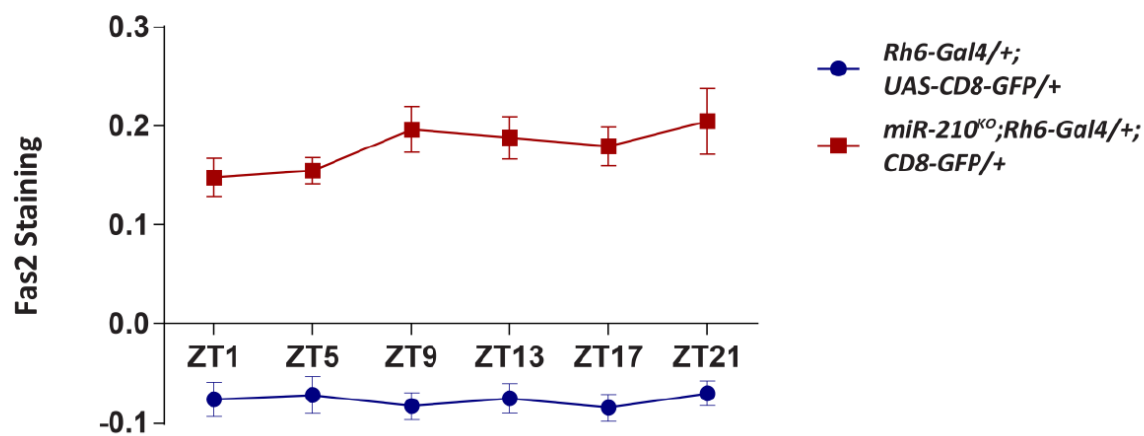

Supplement: S7 Fig — (A) Representative images of fly brains at different time points under LD. Flies were entrained under LD cycles and brains were dissected at the 4th day in LD. Red, Fas2 staining, green, GFP. Rh6-Gal4 was used to label R8 positive photoreceptors and H-B eyelet. Note the increase of Fas2 is found in the optic lobe of miR-210KO at all six time points. Scale bar is 10 μm. (B) Quantification of Fas2 in the optic lobe of wild-type and miR-210KO. Fas2 is constantly higher in the miR-210KO mutant but does not show an oscillation. Fas2 abundance is quantified as Fig 5C. (PDF) [file pgen.1007655.s007.pdf]
