# Supplementary material for: miR-210 controls the evening phase of circadian locomotor rhythms through repression of Fasciclin 2
Source: PLoS Genet. 2019 Jul 29;15(7):e1007655. doi: 10.1371/journal.pgen.1007655 (PMC6687186; doi:10.1371/journal.pgen.1007655)
Supplement: S1 Table — The flybase number of the lines used here are: 58880 (mir-10), 58885 (mir-1007), 58888 (mir-1014), 58889 (mir-1017), 58890 (mir-11), 58892 (mir-133), 58894 (mir-13b-2), 58896 (mir-184), 58898 (mir-193), 58899 (mir-210), 58900 (mir-219), 58904 (mir-274), 58908 (mir-277-34), 58909 (mir-278), 58910 (mir-281-1-281-2), 58911 (mir-282), 58912 (mir-283), 58913 (mir-284), 58914 (mir-285), 58915 (mir-2b-1), 58916 (mir-2c-13a-1), 58917 (mir-303), 58924 (mir-314), 58926 (mir-317), 58928 (mir-31a), 58929 (mir-31b), 58930 (mir-33), 58892 (mir-133), 58935 (mir-927), 58937 (mir-92a), 58939 (mir-932), 58940 (mir-955), 58942 (mir-957), 58943 (mir-958), 58948 (mir-967), 58949 (mir-968-1002), 58952 (mir-971), 58953 (mir-971-972-973), 58954 (mir-975-976-977), 58959 (mir-986), 58960 (mir-987), 58961 (mir-988), 58962 (mir-989), 58963 (mir-990), 58966 (mir-999), 58960 (mir-987), 58967 (mir-9c). (DOCX) [file pgen.1007655.s008.docx]

**Table S1:** Locomotor activity of miR[KO] flis in DD

| Table1: Locomotor activity of flies in DD | | | | |
| --- | --- | --- | --- | --- |
| Genotype | N | % Rhythmic | Period (hr) ± S.E.M. | Power* ± S.E.M |
| *Iso^31^*  *w^1118^*  *yw*  *mir-10[KO] mir-1007[KO] mir-1014[KO] mir-1017[KO] mir-11[KO.w-] mir-133[KO] mir-13b-2[KO] mir-184[KO] mir-193[KO] mir-210[KO] mir-219[KO] mir-274[KO] Df(3R)mir-277-34-KO mir-278[KO] Df(2R)mir-281-1-281-2-KO mir-282[KO] mir-283[KO] mir-285[KO] mir-2b-1[KO] Df(3R)mir-2c-13a-13b-1-KO mir-303[KO] Df(2R)mir-307a-307b-KO mir-314[KO] mir-317[KO] mir-31a[KO] mir-31b[KO] mir-33[KO] mir-133[KO] mir-927[KO] mir-92a[KO] mir-932[KO] mir-955[KO] mir-957[KO] mir-958[KO] mir-967[KO] Df(2L)mir-968-1002-KO mir-971[KO] Df(1)mir-972-973-974-KO Df(1)mir-975-976-977-KO mir-986[KO] mir-987[KO] mir-988[KO] mir-989[KO] mir-990[KO] mir-999[KO] mir-9c[KO]* | 51  48  31  45 23 34 43 24 19 30 22 30 47 19 22 30 42 30 23 23 29 20 24  45 41 23 28 24 27 30 31 44 24 18 25 26 30 22 27 26 28 25 23 30 32 28 40 29 39 | 97.4±1.7  98.3±1.1  98.6±1.0  82.2±2.2 91.3±1.7 82.4±2.5 90.7±1.3 62.5±4.8 84.2±3.1 80.0±2.9 95.5±0.9 80.0±2.9 83.0±2.1 84.2±3.1 81.8±3.2 76.7±3.3 95.2±0.7 86.7±2.1 91.3±1.7 100.0±0.0 72.4±3.7 85.0±2.9 70.8±4.2  91.1±1.2 95.1±0.7 65.2±4.7 60.7±4.5 83.3±2.8 88.9±1.9 90.0±1.6 90.3±1.6 77.3±2.6 87.5±2.2 94.4±1.2 68.0±4.4 92.3±1.4 76.7±3.3 90.9±1.8 85.2±2.4 92.3±1.4 82.1±2.8 92.0±1.5 83.4±5.5 93.3±1.1 93.8±1.0 85.7±2.3 90.0±1.4 72.4±3.7 84.6±1.5 | 24.4±0.2  24.2±0.3  24.6±0.2  24.1±0.3 25.0±0.1 24.6±0.3 24.6±0.2 24.2±0.1 25.0±0.3 23.9±0.2 24.8±0.3 24.4±0.2 24.3±0.1 24.5±0.8 24.3±0.3 24.8±0.1 24.5±0.3 24.2±0.0 24.2±0.2 24.0±0.2 24.4±0.4 23.9±0.3 24.9±0.3  24.5±0.3 24.9±0.2 24.2±0.1 24.5±0.4 23.7±0.1 24.6±0.0 24.3±0.0 24.7±0.1 24.3±0.2 24.1±0.3 24.5±0.1 24.8±0.2 24.4±0.5 24.5±0.4 24.9±0.3 24.2±0.1 24.5±0.4 24.5±0.1 24.5±0.1 24.1±0.2 24.7±0.2 24.0±0.2 24.6±0.2 24.4±0.3 24.1±0.0 24.5±0.4 | 98.2±1.4  99.6±1.1  98.7±1.8  86.1±3.4 92.6±07.6 73.4±4.3 76.9±8.2 86.0±6.4 85.2±3.2 72.5±9.3 80.4±5.5 74.2±8.7 87.4±4.6 93.2±2.4 85.3±5.3 87.2±2.7 91.4±1.3 85.6±4.3 88.1±7.2  103.3±2.2 74.1±3.4 73.0±3.7 72.9±4.7  90.9±3.2 94.5±1.1 62.4±10.2 75.1±4.6 84.0±2.6 83.9±7.0 84.5±5.6 86.9±8.1 73.2±4.8 84.8±6.3 92.7±3.2 74.7±5.8 90.3±4.5 79.8±6.4 88.0±3.7 86.2±3.1 90.1±6.6 84.2±9.0 86.2±4.2 88.5±6.1 90.2±2.7 92.8±1.7 86.3±8.6 87.2±4.5 77.5±4.6 86.8±4.7 |
|  |  |  |  |  |

*Power is a measure of rhythm amplitude and corresponds to the height of the periodogram peak above the significance line.
