# Supplementary material for: miR-210 controls the evening phase of circadian locomotor rhythms through repression of Fasciclin 2
Source: PLoS Genet. 2019 Jul 29;15(7):e1007655. doi: 10.1371/journal.pgen.1007655 (PMC6687186; doi:10.1371/journal.pgen.1007655)
Supplement: S2 Table — (DOCX) [file pgen.1007655.s009.docx]

| Table 1: Locomotor activity of flies in constant temperature | | | | | |
| --- | --- | --- | --- | --- | --- |
| Genotype | N | % Rhythmic | Period (hr) ±S.E.M. | Power (hr) ±S.E.M. | Temperature  (℃) |
| *miR-210^KO^* | 43 | 93.3±5.6 | 23.7±0.1 | 79.8±2.8 | 21 |
| *yw* | 40 | 89.2±8.2 | 23.4±0.1 | 63.7±3.5 | 21 |
| *miR-210^KO^;;*  *UAS-miR-210^KO^* | 34 | 75.5±16.2 | 23.8±0.1 | 65.2±4.1 | 21 |
| *miR-210^KO^* | 32 | 86.7±10.9 | 23.9±0.1 | 68.1±4.8 | 29 |
| *yw* | 44 | 97.8±3.2 | 24.2±0.1 | 81.2±2.6 | 29 |
| *miR-210^KO^;;*  *UAS-miR-210^KO^* | 35 | 79.9±22.3 | 23.9±0.2 | 68.3±8.9 | 29 |
